# Unveiling unified patterns in Alzheimer’s disease subtypes: An SCCA clustering approach integrating PET imaging and genomics data

**DOI:** 10.1162/IMAG.a.1151

**Published:** 2026-02-25

**Authors:** Fan Yang, Matthew Maher, Richa Saxena, Joyita Dutta

**Affiliations:** Department of Biomedical Engineering, University of Massachusetts Amherst, Amherst, MA, United States; Center for Genomic Medicine, Massachusetts General Hospital, Boston, MA, United States; Department of Anesthesia, Critical Care and Pain Medicine, Massachusetts General Hospital, Boston, MA, United States

**Keywords:** Alzheimer’s disease, imaging genetics, PET, tau, Aβ, sparse canonical correlation analysis

## Abstract

Alzheimer’s disease (AD) is the most common cause of dementia and a significant public health challenge. AD is characterized by the formation of tau and beta-amyloid (Aβ) protein aggregates in the brain, which can be imaged in vivo using positron emission tomography (PET). Integrating genetic and neuroimaging data using imaging genetics tools offers the potential to better understand disease mechanisms and risk factors in this heterogeneous disorder. Here, we present a framework based on Sparse Canonical Correlation Analysis (SCCA) integrated with clustering to identify AD subtypes from PET and genomic data. The SCCA clustering method was applied to tau PET scans (N = 541), Aβ PET scans (N = 907), and corresponding genomics data from the Alzheimer’s Disease Neuroimaging Initiative database. Test-retest studies were used to compare two different SCCA implementations, and longitudinal data were used to assess the stability of the subtyping approach. We identified four tau subtypes and two Aβ subtypes with distinct spatial deposition patterns, consistent with prior imaging studies. Genetic profiles associated with each subtype showed enrichment of specific biological pathways. Our findings suggest that SCCA clustering can help reveal biologically meaningful subtypes of AD. A clearer understanding of AD subtypes could ultimately improve AD diagnosis, prognosis, and treatment strategies.

## 1 Introduction

Alzheimer’s disease (AD) is a progressive neurological disease and one of the leading causes of dementia worldwide (Alzheimer’s Association, 2024). As the population continues to age, the prevalence of AD is rising, creating a pressing necessity for better diagnostic and treatment strategies. The heterogeneity of AD, reflected in its varied clinical symptoms, neuropathological characteristics, and genetic factors, poses a considerable challenge for the development of universally effective treatments (Duara et al., 2022; Holtzman et al., 2011; Jack et al., 2018). Hallmarks of AD include the accumulation of extracellular beta-amyloid (Aβ) plaques and intracellular tau tangles, both of which can now be measured in vivo using positron emission tomography (PET) imaging (Chien et al., 2013; Klunk et al., 2004). Early studies using tau PET imaging revealed spatial patterns of tau pathology (Cho et al., 2016; Schöll et al., 2016; Schwarz et al., 2016) that show a strong correspondence with the Braak staging system (Braak & Braak, 1991; Braak et al., 2006). Similarly, Aβ PET studies have explored how patterns of Aβ deposition relate to Braak staging in vivo, using both classification-based methods and data-driven analyses (Grothe et al., 2017; Jelistratova et al., 2020; Mattsson et al., 2019).

While the Braak staging system provides a foundation structure for describing tau progression in AD, it does not account for the considerable variability observed at the individual level (Vogel et al., 2021). Our own studies have demonstrated that tau propagation can be modeled at the individual level to capture specific trajectories rather than assuming a uniform global sequence (Song et al., 2020; F. Yang et al., 2019, 2021). Such heterogeneity may manifest clinically in distinct syndromes, for example posterior cortical atrophy (Crutch et al., 2017; Ossenkoppele et al., 2016). Identifying AD subtypes could, therefore, improve diagnostic accuracy and prognostic precision (Arafah et al., 2023; Drummond et al., 2017). In recent years, AD subtyping has become an increasingly active area of research. Several studies have proposed subtypes using postmortem data, magnetic resonance imaging (MRI), tau PET, and Aβ PET (Ferreira et al., 2020). Early autopsy-based research classified AD into typical, hippocampal-sparing, and limbic-predominant subtypes by evaluating the density and distribution of neurofibrillary tangles in cortical and hippocampal regions (Murray et al., 2011; Whitwell et al., 2012). A fourth subtype, minimal atrophy AD, has been consistently identified in the literature, characterized by a lack of pronounced regional atrophy (Persson et al., 2017; Shima et al., 2012). Data-driven MRI studies have further refined atrophy subtypes in mild AD, categorizing it into medial temporal, parietal-dominant, and diffuse atrophy using Ward’s clustering on cortical thickness (Habes et al., 2020; Noh et al., 2014). The Subtype and Stage Inference (SuStaIn) model, developed to reveal subtypes with common progression patterns using cross-sectional biomarker datasets, has further advanced this field (Young et al., 2018). Four tau PET subtypes, namely limbic, medial temporal lobe (MTL)-sparing, posterior, and lateral temporal (Vogel et al., 2021), and two Aβ PET subtypes, namely cortex-priority and subcortex-priority (Sun et al., 2023), have been identified using SuStaIn. Although these subtypes provide meaningful insights into disease mechanisms, their dependence on phenotype-based data alone limits their ability to fully characterize the genetic complexity underlying AD.

AD is a genetically complex disorder, with both rare and common genetic variants contributing to its development (Bellenguez et al., 2022; Fortea et al., 2024; Frisoni et al., 2022). The early-onset autosomal dominant form of AD is caused by mutations in three primary genes: APP, PSEN1, and PSEN2 (Frisoni et al., 2022). In contrast, the more common sporadic or late-onset AD (LOAD) has been linked to a broader range of genetic variants. Among these, the APOE gene is the strongest and most well-established risk factor for sporadic AD (Naslavsky et al., 2022). However, genetic studies on LOAD have highlighted the heterogeneous nature of the disease, with dozens of other genetic variants also implicated in increasing AD risk. Over the years, genome-wide association studies (GWAS) have identified numerous risk loci associated with AD (Jansen et al., 2022; Kondo et al., 2022; Lambert et al., 2013; Wightman et al., 2021). The most recent two-stage GWAS has revealed a much larger pool of over 70 such loci (Bellenguez et al., 2022). These loci include genes such as ABCA7, PICALM, BIN1, CLU, PLCG2, SORL1, and TREM2, many of which have rare coding variants that influence the disease (Sims et al., 2017). The genes identified from these studies suggest that AD pathogenesis is mediated by multiple biological pathways, spanning processes like lipid metabolism, immune response, and Aβ clearance (Young-Pearse et al., 2023). This genetic diversity suggests that AD subtypes may reflect heterogeneity in their genetic architecture, reinforcing the potential for personalized therapeutic strategies that focus on pathways relevant to individual disease progression (Freudenberg-Hua et al., 2018).

Recent advances in imaging genetics have provided new opportunities to explore the complex and varied manifestations of AD (Hariri & Weinberger, 2003). By combining neuroimaging methods such as PET and MRI with genetic information, researchers can examine how imaging-derived phenotypic signatures and related genetic variants influence spatial patterns of neurodegeneration (Sims et al., 2020; Yu et al., 2021). This approach has the potential to characterize genotype-phenotype relationships in AD (Arafah et al., 2023; Saykin et al., 2010). Previous research in imaging genetics has employed a range of computational approaches, including canonical correlation analysis (CCA), partial least squares regression, and sparse reduced-rank regression, to associate genetic variants with regions-of-interest (ROIs) in the brain (Geladi & Kowalski, 1986; Hardoon & Shawe-Taylor, 2011; Vounou et al., 2010). These methods have helped identify risk genes and their corresponding brain regions, supporting an understanding of the relationship between genetic risk factors and spatial pathological signatures (Xin et al., 2022). Additionally, researchers have refined correlation analysis methods to improve interpretability and reduce overfitting, with sparse models proving particularly useful (Fu et al., 2017; Mai & Zhang, 2019). In the context of AD subtyping, imaging genetics offers the opportunity to further characterize disease’s heterogeneity (Elman et al., 2024). By correlating genetic variants with specific AD subtypes, we can examine how genetic factors contribute to distinct patterns of tau or Aβ aggregation. To our knowledge, no existing framework has combined PET imaging with genetic data for subtyping, resulting in a lack of direct connection between spatial patterns of tau and Aβ deposition and genetic variation. Addressing this gap is important for identifying biologically grounded subtypes that link molecular mechanisms with imaging trajectories. Sparse Canonical Correlation Analysis (SCCA) is a suitable method for jointly analyzing genetic and imaging data in AD, as it can capture relationships between high-dimensional datasets while promoting sparsity for robustness. In combination with clustering, SCCA has the potential to identify AD subtypes from joint imaging and genetic patterns. Beyond their value for characterizing AD heterogeneity, genetically informed subtypes may also have translational potential by improving diagnosis, informing prognosis, and guiding personalized treatment strategies.

In this paper, we present a novel subtyping strategy that leverages SCCA combined with clustering in a unified framework that uses PET imaging and genomic data for the identification of AD subtypes. This study examines AD subtypes that are defined not only by patterns of tau or Aβ deposition, but also by associated genetic variation. In Section 2, we derive an algorithm for the SCCA clustering framework and provide data descriptions and processing details. Our primary findings, presented in Section 3, demonstrate the algorithm’s convergence and reliability for tau and Aβ PET data and include PET-based spatial profiles and single-nucleotide polymorphisms (SNPs) dosage profiles for different subtypes identified using our method. Finally, in Sections 4 and 5, we summarize our work, discuss its strengths and limitations, and outline our envisioned future directions.

## 2 Method

### 2.1 SCCA clustering

**Fig. 1.**
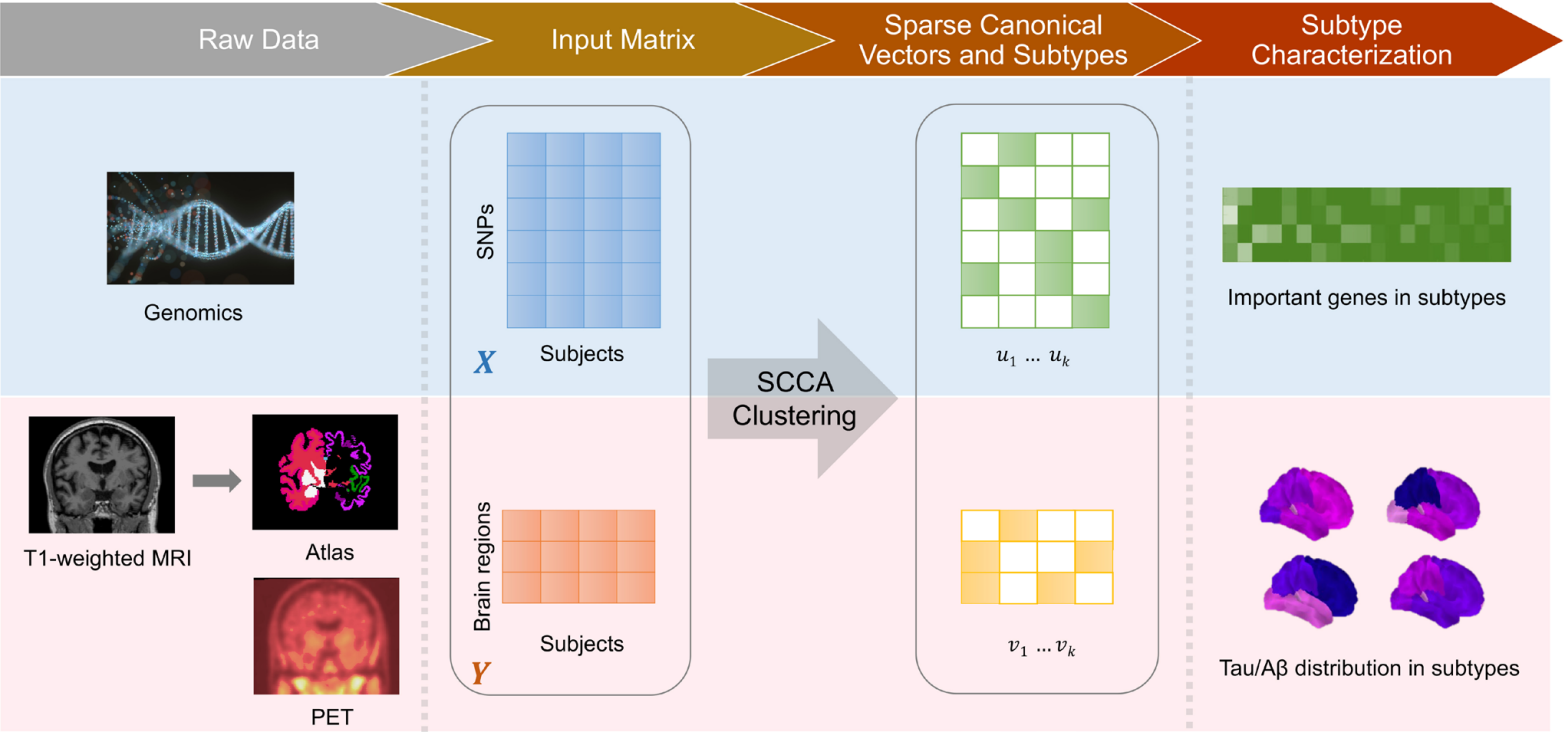
Overview of the workflow illustrating the SCCA clustering process, showing the sample genomic data and imaging data, including MRI-derived ROI segmentations and PET scans. Genomic data are processed by calculating the dosage of the risk alleles, with results represented as vectors for each subject. The T1-weighted MRI scans are segmented using FreeSurfer to generate region-wise PET vectors. SCCA clustering yields subtype-specific sparse canonical vectors, depicted as green and yellow matrices. For each subtype, the average genomic dosage is ranked to identify significant patterns, while the average PET data is visualized on brain surface plots.

In this section, we first present a CCA clustering framework that combines CCA and clustering via an integrated algorithm with the objective of simultaneously uncovering meaningful correlations between two distinct data modalities and grouping subjects based on shared patterns. By iteratively optimizing correlation structures, this approach not only identifies latent relationships within the data but also enhances cluster formation. Building upon this framework, SCCA clustering, illustrated in Figure 1, introduces additional sparsity constraints on the canonical vectors to refine the CCA objective function (Hardoon & Shawe-Taylor, 2011). We investigate two constraint-based models, namely Iterative Penalized Least Squares (IPLS) SCCA and Elastic SCCA (Fu et al., 2017; Mai & Zhang, 2019).

#### 2.1.1 CCA clustering

##### 2.1.1.1 Cost function and membership matrix

We denote the genetic data as $\text{X}\in {\mathbb{R}}^{M\times P}$ and the PET data as $\text{Y}\in {\mathbb{R}}^{M\times N}$ . Here, M denotes the number of subjects, N represents the number of ROIs, and P signifies the number of SNPs. The CCA cost function is defined as:

$$J=(\text{Xu}{)}^{\prime }\text{Yv},$$ (1)

where $\text{u}\in {\mathbb{R}}^{P\times 1}$ , $\text{v}\in {\mathbb{R}}^{N\times 1}$ . This cost function can be reformulated by introducing intermediate variables $\alpha_{i}$ and $\beta_{i}$, leading to:

$$J=\sum_{i=1}^{M}\alpha_{i}\beta_{i},$$ (2)

where

$$\alpha =\text{Xu},\;\;\alpha_{i}=\sum_{j=1}^{P}u_{j}X_{ij},$$ (3)

$$\beta =\text{Yv},\;\;\beta_{i}=\sum_{j=1}^{N}v_{j}Y_{ij}.$$ (4)

To incorporate simultaneous clustering, we introduce a binary membership matrix $\text{R}\in {\mathbb{R}}^{M\times K}$ , where K denotes the number of clusters. Each column of R is a vector ${\text{r}}_{k}\in {\mathbb{R}}^{M\times 1}$ , with entries indicating cluster membership. Using ${\text{r}}_{k}$, we define diagonal matrices ${\text{D}}_{k}={\text{diag}}_{k}({\text{r}}_{k})$ $\in {\mathbb{R}}^{M\times M}$ for scaling X and Y. This leads to the modified cost function:

$$J=\sum_{k=1}^{K}{\left(D_{k}Xu_{k}\right)}^{\prime }D_{k}Yv_{k}=\sum_{k=1}^{K}u_{k}^{\;\;\;\;\prime }X^{\prime }D_{k}^{\;\;\;\prime }D_{k}Yv_{k}=\sum_{k=1}^{K}\sum_{I\in M_{k}}\alpha_{ik}\beta_{ik}$$ (5)

where ${ℳ}_{k}$ represents the set of subjects belonging to the k-th cluster. Here, $\alpha_{ik}$ and $\beta_{ik}$ are intermediate variables defined as:

$$\alpha_{k}=\text{X}{\text{u}}_{k},\alpha_{ik}=\sum_{j=1}^{P}u_{jk}X_{ij},$$ (6)

$$\beta_{k}=\text{Y}{\text{v}}_{k},\beta_{ik}=\sum_{j=1}^{N}v_{jk}Y_{ij}.$$ (7)

##### 2.1.1.2. Cluster membership assignment and canonical vector updates

To assign subjects to clusters, we compute the cluster membership by maximizing the product of $\alpha_{ik}$ and $\beta_{ik}$ for each subject i:

$${\hat{k}}_{i}=\text{arg}\;\underset{k}{\;\text{max}}\;\alpha_{ik}\beta_{ik}.$$ (8)

This can alternatively be formulated by minimizing the squared difference:

$${\hat{\text{u}}}_{k},{\hat{\text{v}}}_{k}=\text{arg }\underset{k}{\text{min}}∥\alpha_{ik}-\beta_{ik}{∥}^{2}.$$ (9)

After determining memberships, the canonical vectors ${\text{u}}_{k}$ and ${\text{v}}_{k}$ are updated by solving:

$${\hat{\text{u}}}_{k},{\hat{\text{v}}}_{k}=\text{arg }\underset{{\text{u}}_{k},{\text{v}}_{k}}{\;\text{max}\;}\alpha_{ik}\beta_{ik},$$ (10)

or equivalently by solving:

$${\hat{\text{u}}}_{k},{\hat{\text{v}}}_{k}=\arg\;\underset{u_{k},v_{k}}{\;\text{min}}∥\alpha_{ik}-\beta_{ik}{∥}^{2}\;.$$ (11)

##### 2.1.1.3. Final membership matrix update

Once ${\text{u}}_{k}$ and ${\text{v}}_{k}$ are updated, the binary membership matrix R is recalculated as:

$$R_{ik}=\{\begin{array}{cc} 1 & \text{if }k={\hat{k}}_{i},\\ 0 & \text{otherwise}. \end{array}\;$$ (12)

#### 2.1.2 IPLS SCCA clustering

In the IPLS SCCA clustering framework, we use iterative re-scaled LASSO regression to enforce sparsity. This iterative process refines the CCA cost by identifying and emphasizing significant correlations in the data (Mai & Zhang, 2019). The modified IPLS cost function is defined as:

$$J=\;∥\text{Xu}-\text{Yv}{∥}^{2}+\;\lambda_{u}∥\text{u}{∥}_{1}+\;\lambda_{v}∥\text{v}{∥}_{1},$$ (13)

where $\lambda_{u},\lambda_{v}>0$ are tuning parameters. When combined with clustering, the IPLS objective becomes:

$$J_{IPLS}=\sum_{k=1}^{K}\left(∥{\text{D}}_{k}\text{X}{\text{u}}_{k}-{\text{D}}_{k}\text{Y}{\text{v}}_{k}\;{∥}^{2}\;+\;\lambda_{u}\;∥\text{u}{∥}_{1}+\;\lambda_{v}\;∥\text{v}{∥}_{1}\right),$$ (14)

The following steps describe the process of determining the optimal membership and updating cluster-specific variables.

1. Cluster membership assignment, update R: For each subject and each cluster, compute:

$${\hat{k}}_{i}=\text{arg}\;\underset{k}{\text{min}}∥{\text{X}}_{i}{\hat{\text{u}}}_{k}-{\text{Y}}_{i}{\hat{\text{v}}}_{k}{∥}^{2}+\;\lambda_{u}∥{\hat{\text{u}}}_{k}{∥}_{1}+\;\lambda_{v}∥{\hat{\text{v}}}_{k}{∥}_{1}.$$ (15)

2. Update canonical vectors ${\text{u}}_{k}$ and ${\text{v}}_{k}$:

$${\hat{\text{u}}}_{k},{\hat{\text{v}}}_{k}=\underset{{\text{u}}_{k},{\text{v}}_{k}}{\text{min}}\;J_{IPLS}.$$ (16)

#### 2.1.3 Elastic SCCA clustering

Elastic CCA is an extension of traditional CCA that incorporates elastic net regularization, a hybrid penalty function combining L1 (LASSO) and L2 (Ridge) regularization, to enhance feature selection and reduce overfitting while discovering complex relationships between multivariate datasets (Fu et al., 2017). The cost can be presented as:

$$J=\;∥\text{Xu}-\text{Yv}{∥}^{2}+\;\lambda_{u}∥\text{u}{∥}_{1}+\;\lambda_{v}∥\text{v}{∥}_{1}+\;\gamma_{u}∥\text{u}{∥}_{2}^{2}+\;\gamma_{v}∥\text{v}{∥}_{2}^{2},$$ (17)

where $\lambda_{u},\lambda_{v},\gamma_{u},\gamma_{v}>0$ are tuning parameters. For Elastic SCCA clustering, the clustering cost is given by:

$$\begin{array}{l} J_{Elastic}=\sum_{k=1}^{K}(∥{\text{D}}_{k}\text{X}{\text{u}}_{k}-{\text{D}}_{k}\text{Y}{\text{v}}_{k}\;{∥}^{2}\;+\;\lambda_{u}\;∥\text{u}{∥}_{1}+\;\lambda_{v}∥\text{v}{∥}_{1}\\ \;\;\;\;\;\;\;\;\;\;\;\;\;\;\;\;\;\;\;\;\;+\;\gamma_{u}\;∥\text{u}{∥}_{2}^{2}+\;\gamma_{v}∥\text{v}{∥}_{2}^{2}). \end{array}$$ (18)

Similar to IPLS, the steps for updating the canonical vectors and assigning memberships are as follows:

1. Cluster membership assignment, update R: For each subject and each cluster, compute:

$$\begin{array}{l} {\hat{k}}_{i}=\text{arg}\;\underset{k}{\text{min}}∥{\text{X}}_{i}{\hat{\text{u}}}_{k}-{\text{Y}}_{i}{\hat{\text{v}}}_{k}\;{∥}^{2}+\;\lambda_{u}∥{\hat{\text{u}}}_{k}\;{∥}_{1}+\;\lambda_{v}\;∥{\hat{\text{v}}}_{k}\;{∥}_{1}\\ \;\;\;\;\;\;\;\;\;\;+\;\gamma_{u}\;∥{\hat{\text{u}}}_{k}\;{∥}_{2}^{2}\;+\;\gamma_{v}\;∥{\hat{\text{v}}}_{k}\;{∥}_{2}^{2} \end{array}$$ (19)

2. Update canonical vectors ${\text{u}}_{k}$ and ${\text{v}}_{k}$:

$${\hat{\text{u}}}_{k},{\hat{\text{v}}}_{k}=\underset{{\text{u}}_{k},{\text{v}}_{k}}{\text{min }}\;J_{Elastic}.$$ (20)

The pseudo-code of the SCCA clustering algorithm is shown as Algorithm 1.

**Algorithm 1:** SCCA Clustering (for IPLS and Elastic)

**Input:** Genetic data $\text{X}\in {\mathbb{R}}^{M\times P}$ (P SNPs), PET imaging data $\text{Y}\in {\mathbb{R}}^{M\times N}$ (M subjects, N ROIs), Tuning parameters $\lambda_{u},\lambda_{v}$ (and $\gamma_{u},\gamma_{v}$ for Elastic), Number of clusters K. **Output:** Membership matrix R, canonical vectors ${\text{u}}_{k},{\text{v}}_{k}$ for each cluster k. Initialize u,v for the entire cohort and randomly perturb the initialization. Set ${\text{u}}_{k}$ and ${\text{v}}_{k}$ for each cluster k based on the initial u and v. **repeat** **for** each subject i=1,…,M **do** Update membership R: Assign subject i to the cluster k that minimizes: ${\hat{k}}_{i}=\text{arg}\;\underset{k}{\text{min}}\left(∥{\text{X}}_{i}{\hat{\text{u}}}_{k}-{\text{Y}}_{i}{\hat{\text{v}}}_{k}\;{∥}^{2}\;+\;\lambda_{u}\;∥{\hat{\text{u}}}_{k}\;{∥}_{1}+\;\lambda_{v}\;∥{\hat{\text{v}}}_{k}\;{∥}_{1}+\;\gamma_{u}\;∥{\hat{\text{u}}}_{k}\;{∥}_{2}^{2}\;+\;\gamma_{v}\;∥{\hat{\text{v}}}_{k}\;{∥}_{2}^{2}\right).$ **end for** **for** each cluster k=1,…,K **do** Update ${\text{u}}_{k},{\text{v}}_{k}$: Optimize the objective function for the cluster k: $J_{k}=\sum_{i\in {ℳ}_{k}}\left(∥{\text{X}}_{i}{\text{u}}_{k}-{\text{Y}}_{i}{\text{v}}_{k}\;{∥}_{2}^{2}\;+\;\lambda_{u}\;∥{\text{u}}_{k}\;{∥}_{1}+\;\lambda_{v}\;∥{\text{v}}_{k}\;{∥}_{1}\;+\;\gamma_{u}\;∥{\text{u}}_{k}\;{∥}_{2}^{2}\;+\;\gamma_{v}\;∥{\text{v}}_{k}\;{∥}_{2}^{2}\right).$ For IPLS: Set $\gamma_{u}=0,\gamma_{v}=0$ . For Elastic: Include both L1 and L2 regularization terms. **end for** **until** convergence **Return:** $\text{R},{\text{u}}_{k},{\text{v}}_{k}$

### 2.2 Data description

#### 2.2.1 Participants

The data utilized in this article were sourced from the Alzheimer’s Disease Neuroimaging Initiative (ADNI) database, a multisite study that acquired neuroimaging, neuropsychological, biochemical, genetic, and other measurements from cognitively normal and impaired individuals (http://adni.loni.usc.edu/). Specifically, our study leveraged data from ADNI2 or ADNI3 participants with available genetic data containing AD-related SNPs, who underwent PET scans based on the ^18^F-Flortaucipir radiotracer for tau (N = 541) or the ^18^F-Florbetapir radiotracer for Aβ (N = 907). Among these participants, 207 individuals from the tau group and 597 from the Aβ group had multiple PET scans conducted at different time-points. All participants had a clinical diagnostic status of cognitively normal (CN), mild cognitive impairment (MCI), or AD. A summary of participant characteristics is provided in Table 1.

**Table 1.** Participant demographics.*

	Tau				Aβ			
	All	CN	MCI	AD	All	CN	MCI	AD
Demographics								
N	541	334	174	33	907	344	432	131
Sex (% female)	47.69%	40.12%	59.77%	60.61%	52.26%	44.19%	56.48%	59.54%
Age^†^ (yrs.)	73.87 ± 7.30	73.56 ± 7.04	74.40 ± 7.40	74.15 ± 9.17	73.99 ± 7.23	74.61 ± 6.42	73.31 ± 7.31	74.62 ± 8.70
Education (yrs.)	16.60 ± 2.45	16.83 ± 2.34	16.30 ± 2.54	15.91 ± 2.75	16.31 ± 2.57	16.71 ± 2.45	16.18 ± 2.60	15.71 ± 2.64
Cognition								
MMSE^‡^	28.24 ± 2.31	29.10 ± 0.96	27.67 ± 2.06	22.63 ± 3.94	27.12 ± 3.09	28.89 ± 1.03	27.36 ± 2.19	21.68 ± 3.06
Memory	0.77 ± 0.86	1.15 ± 0.61	0.35 ± 0.76	-0.78 ± 0.65	0.41 ± 0.98	1.07 ± 0.62	0.31 ± 0.83	-0.99 ± 0.53
Executive function	0.77 ± 0.91	1.08 ± 0.74	0.46 ± 0.80	-0.69 ± 1.00	0.32 ± 1.06	0.86 ± 0.76	0.31 ± 0.91	-1.09 ± 0.90
Visuospatial function	0.12 ± 0.61	0.25 ± 0.52	0.01 ± 0.59	-0.54 ± 0.96	-0.06 ± 0.69	-0.19 ± 0.48	-0.04 ± 0.60	-0.78 ± 0.94
Language	0.66 ± 0.80	0.96 ± 0.62	0.34 ± 0.72	-0.66 ± 0.89	0.31 ± 0.97	0.84 ± 0.65	0.28 ± 0.81	-0.99 ± 0.92

* Values are in the format mean ± standard deviation.

† Age at the time of PET scan.

‡ MMSE: Mini-Mental State Examination.

#### 2.2.2 Image data acquisition and processing

PET standardized uptake value ratios (SUVRs) were derived from the UC Berkeley processed datasets from ADNI. A full description of the acquisition and processing procedures for ^18^F-Flortaucipir and ^18^F-Florbetapir PET scans can be found in the ADNI UC Berkeley methods documents. Regional PET measurements were obtained for all ROIs defined by FreeSurfer, utilizing MRI scans obtained closest in time to each PET scan. SUVRs were computed using the whole cerebellum for Aβ PET and the inferior cerebellar gray matter for tau PET. These measurements were downloaded from ADNI, and, subsequently, mean SUVRs were extracted based on significant spatial characteristics (10 ROIs for tau PET and 11 ROIs for Aβ PET). Lastly, we expressed each regional PET measurement as a z-score using the entire cohort to ensure consistent normalization across all individuals. This cohort-wide normalization is critical for the joint PET-genetic SCCA clustering framework, as it maintains comparability between subjects without introducing group-specific reference baselines. For tau PET, we analyzed the MTL, parietal, frontal, occipital, and temporal lobes in both hemispheres, along with their corresponding subregions (listed in Supplementary Table S1). For Aβ PET, eleven regions were included: frontal, temporal, parietal, occipital, insula, amygdala, cingulate, hippocampus, thalamus, basal ganglia, and cerebellum. All regions were defined in the first level of the Human Brainnetome atlas (Fan et al., 2016), consistent with prior studies identifying these areas as critical for characterizing tau and Aβ deposition in AD (Sun et al., 2023; Vogel et al., 2021).

#### 2.2.3 Genomics data acquisition and processing

Genotyping of ADNI participants was conducted in accordance with the manufacturer’s protocol, utilizing genomic DNA samples obtained from blood. For ADNI3, the Illumina GWAS array Global Screening Array v2 was employed, while ADNI2 utilized the Human Omni Express array. To ensure data quality, a series of quality control (QC) measures, imputation, and data integration procedures were performed using PLINK v1.90, during which SNPs were excluded when the missing genotype rate exceeded 2% or the Hardy-Weinberg Equilibrium statistic was less than $10^{-6}$ . Imputation was then performed using the TOPMed Imputation server (Minimac4 algorithm using the TOPMed R2 reference panel).

We assembled an extensive set of SNPs associated with AD from multiple published sources, including large-scale GWAS and targeted imaging-genetics studies. SNPs were included if previously reported to show significant associations with clinical AD diagnosis, AD risk, or AD-related endophenotypes such as amyloid/tau burden, hippocampal atrophy, MRI-based neurodegeneration markers, or cognitive decline. This search initially resulted in 236 SNPs. Following QC and imputation, 211 of these SNPs with known AD risk-increasing alleles were successfully identified, and dosages for the AD risk-increasing alleles for the 211 identified SNPs were extracted to facilitate further analyses (the list of 211 SNPs and reference papers are listed in the Supplementary Table S2). Finally, SNP dosage values were standardized using z-score normalization across participants to place all genetic variables on a comparable scale.

### 2.3 Model implementation and validation

We conducted separate imaging genetics analyses for tau and Aβ. In line with established methodologies in the SuStaIn subtyping literature and to enable direct comparison with existing studies as well as evaluate the method’s ability to recover established subtype patterns, we assume four clusters for tau and two for Aβ (Sun et al., 2023; Vogel et al., 2021). The regularization parameters in the SCCA clustering model were set with different strengths, with a weaker sparsity constraint on PET features to preserve complete spatial patterns across the ROIs, and a stronger sparsity constraint on genetic features to select only the most relevant SNPs from the 211 candidates.

**Fig. 2.**
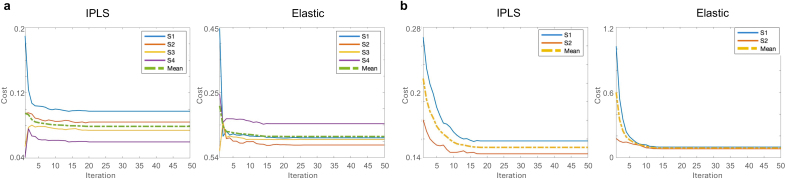
Convergence curves showing the trajectories of the cost function, as defined by Equation 14 for the IPLS model and Equation 18 for the Elastic model, applied to both tau PET (a) and Aβ PET (b) data. Each solid line corresponds to an individual subtyping group, while the dashed line represents the mean value across all groups.

To initialize the clustering, we first computed the canonical vectors for the entire participant cohort. These global vectors were then perturbed in a controlled manner to generate K distinct sets of initial canonical vectors $\left({\text{u}}_{k},{\text{v}}_{k}\right)$ for the predefined number of clusters. This strategy provided diverse and comparable starting points for each cluster, helping reduce sensitivity to initialization and preventing clusters from collapsing or becoming ineffective in the early iterations. With these initializations, we employed an iterative approach to minimize the cost functions, alternately updating cluster membership and sparse canonical vectors until convergence. To monitor optimization behavior, we plot the convergence curves for the IPLS and Elastic SCCA clustering models for tau and Aβ separately. These curves and the corresponding minimum costs are presented in Figure 2 and Table 2. The four tau subtypes are identified as S1, S2, S3, and S4, while the two Aβ subtypes are identified as S1 and S2. Global tau and Aβ SUVR distributions across subtypes are shown in Supplementary Figure S1. The largely overlapping distributions suggest that clustering was driven by regional signal patterns rather than global signal intensity.

**Table 2.** Cost function values of IPLS and elastic models for tau and Aβ PET across subtypes.

	Tau				Aβ	
Cost	S1	S2	S3	S4	S1	S2
IPLS	0.097	0.084	0.074	0.060	0.162	0.141
Elastic	0.111	0.089	0.106	0.154	0.097	0.081

### 2.4 Reproducibility and stability analysis

We evaluated the reproducibility of the subtyping framework using two approaches. The first assessed stability across different initializations and bootstrap resampling using single time-point data. The second evaluated consistency of subtype membership across scans in longitudinal datasets. All analyses were performed separately for tau and Aβ PET subtypes, and results were compared between the IPLS and Elastic SCCA models.

#### 2.4.1 Reproducibility under initialization and resampling variability

We examined reproducibility using single time-point data under two sources of variability: different initializations of the canonical vectors and bootstrap resampling. Each analysis involved one hundred repetitions, either by changing the random seed or by generating resampled datasets with replacement, with the full subtyping procedure repeated on each run. ICC was used to evaluate the reproducibility of the canonical weights, while subtype stability was assessed through separate clustering-consistency analyses.

Genetic reproducibility was quantified using intraclass correlation coefficients (ICCs) derived from binarized SNP weight vectors, where non-zero entries were coded as 1 to indicate selection. ICC values were computed using a two-way mixed-effects, consistency, average-measures model (Koo & Li, 2016). As shown in Table 3, ICC values were generally high across both methods. Elastic SCCA produced higher ICCs than IPLS under different initializations for tau subtypes (92.18%-96.82% vs. 83.19%-94.00%) and slightly higher values for Aβ subtypes (all above 99%). With bootstrap resampling, ICCs decreased for both models due to sampling variability, but Elastic SCCA generally maintained higher reproducibility for tau, whereas values for Aβ dropped more substantially, particularly for Elastic SCCA (71.77%-76.05%).

**Table 3.** ICC for reproducibility under initialization variability and bootstrap resampling.

ICC	Tau (4 subtypes)				Aβ (2 subtypes)	
Subtype	S1	S2	S3	S4	S1	S2
Variable initialization						
IPLS	87.12%	87.46%	94.00%	83.19%	99.02%	99.05%
Elastic	96.00%	93.33%	96.82%	92.18%	99.28%	99.09%
Bootstrapping						
IPLS	82.25%	81.99%	85.38%	74.63%	94.44%	94.38%
Elastic	80.25%	81.24%	85.10%	87.15%	71.77%	76.05%

**Fig. 3.**
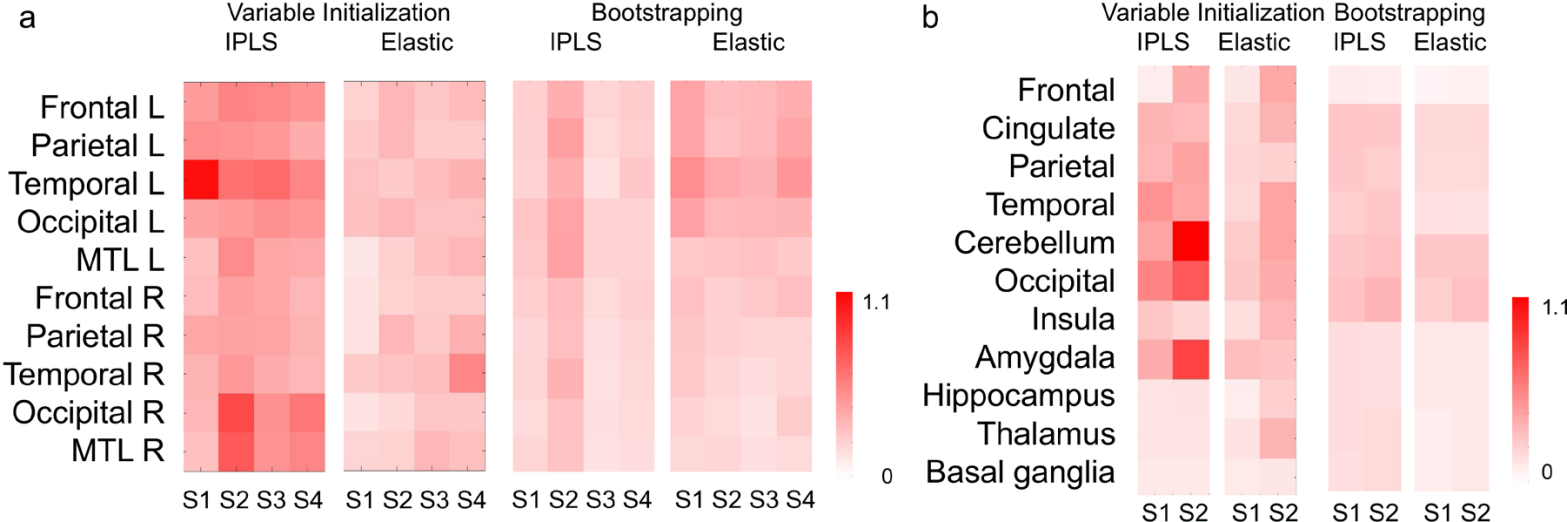
Stability of canonical vectors for tau and Aβ subtypes. Panels show the standard deviation of canonical vectors across 100 repetitions under two evaluation settings: (i) variable initialization and (ii) bootstrap resampling with replacement. Results are displayed for (a) four tau subtypes (S1–S4) across bilateral cortical and medial temporal regions and (b) two Aβ subtypes (S1–S2) across major cortical and subcortical regions. Lower standard deviation indicates higher stability of the canonical vectors.

For imaging features, reproducibility was evaluated by the standard deviation of canonical vector weights across the repetitions under both variable initialization and bootstrap resampling. Lower variability reflects more stable spatial weights across brain regions. As shown in Figure 3, the variability of the canonical vector was reasonably low for both methods. Elastic SCCA exhibited consistently lower and more homogeneous deviations than IPLS for tau and Aβ PET, indicating greater robustness of the estimated canonical vectors.

In addition to evaluating the reproducibility of the canonical weights, we examined the stability of subtype assignments for the Elastic SCCA model under both initialization and resampling variability. Repeated runs with different random initializations produced highly similar subtype structures, with variability mainly among borderline cases (Supplementary Fig. S2a). Bootstrap resampling further confirmed high data level reproducibility (Supplementary Fig. S2b): most subjects were assigned to their predominant subtype in 75% to 95% of runs, and co-assignment probabilities within and between subtypes were consistently high (on the order of 0.7). The binary Aβ grouping showed minimal sensitivity to initialization (mean consistency ≈ 0.99) while exhibiting moderate reproducibility under bootstrap resampling (mean consistency ≈ 0.62).

#### 2.4.2 Longitudinal stability assessment

Longitudinal PET data were available for 207 subjects with tau PET (512 scans) and 597 subjects with Aβ PET (1,838 scans), each with at least two time points. These datasets were used to examine the stability of subtype assignments over time, under the assumption that an individual should remain in the same subtype across repeated scans. All scans from each subject were included in the analysis, and preprocessing and z-scoring were performed in the same way as for the main dataset, using the combined set of all available scans to calculate normalization parameters. Subtype labels were assigned independently at each time-point using the Elastic and IPLS SCCA models, and stability was defined as the proportion of subjects who retained the same subtype across all visits.

As summarized in Table 4, Elastic SCCA showed higher longitudinal consistency than IPLS for tau PET, with 82.52% of subjects remaining in the same subtype across visits compared with 60.68% for IPLS. For Aβ PET, both models produced high stability, with 91.96% for Elastic SCCA and 90.79% for IPLS. These findings indicate that Elastic SCCA provides more consistent subtype assignments in longitudinal settings.

**Table 4.** Multiple time-point consistency for tau and Aβ PET subtypes.

Multiple time-point consistency	Tau (4 subtypes)		Aβ (2 subtypes)	
	IPLS	Elastic	IPLS	Elastic
	60.68%	82.52%	90.79%	91.96%

#### 2.4.3 Integrative assessment of stability and reproducibility

In general, Elastic SCCA showed superior reproducibility between initializations and higher longitudinal consistency, while IPLS demonstrated higher stability under bootstrap resampling. This divergence likely reflects differences in the regularization schemes: Elastic, with its L2 penalty, produces smoother solutions that are less sensitive to initialization and capture more global patterns, thereby improving temporal consistency. IPLS, by enforcing stronger sparsity, emphasizes a smaller set of robust features, which enhances stability under resampling but increases sensitivity to initialization and temporal variation. Considering that reproducibility across initializations and consistency over time are more critical for biological interpretability, the Elastic formulation was adopted as the primary model for subsequent analyses.

## 3 Results

### 3.1 Imaging signatures of subtypes

#### 3.1.1 Subtyping with tau and genetics

We applied Elastic SCCA clustering to tau PET data from 541 participants, predefining four clusters to capture heterogeneity in spatial patterns of tau deposition observed in AD. The model robustly identified four tau subtypes, each characterized by distinct spatial distributions of tau pathology. To quantitatively illustrate these patterns, we computed average regional z-scores of tau PET uptake within each subtype and normalized these values to a 0–1 range, enhancing comparability across subtypes.

**Fig. 4.**
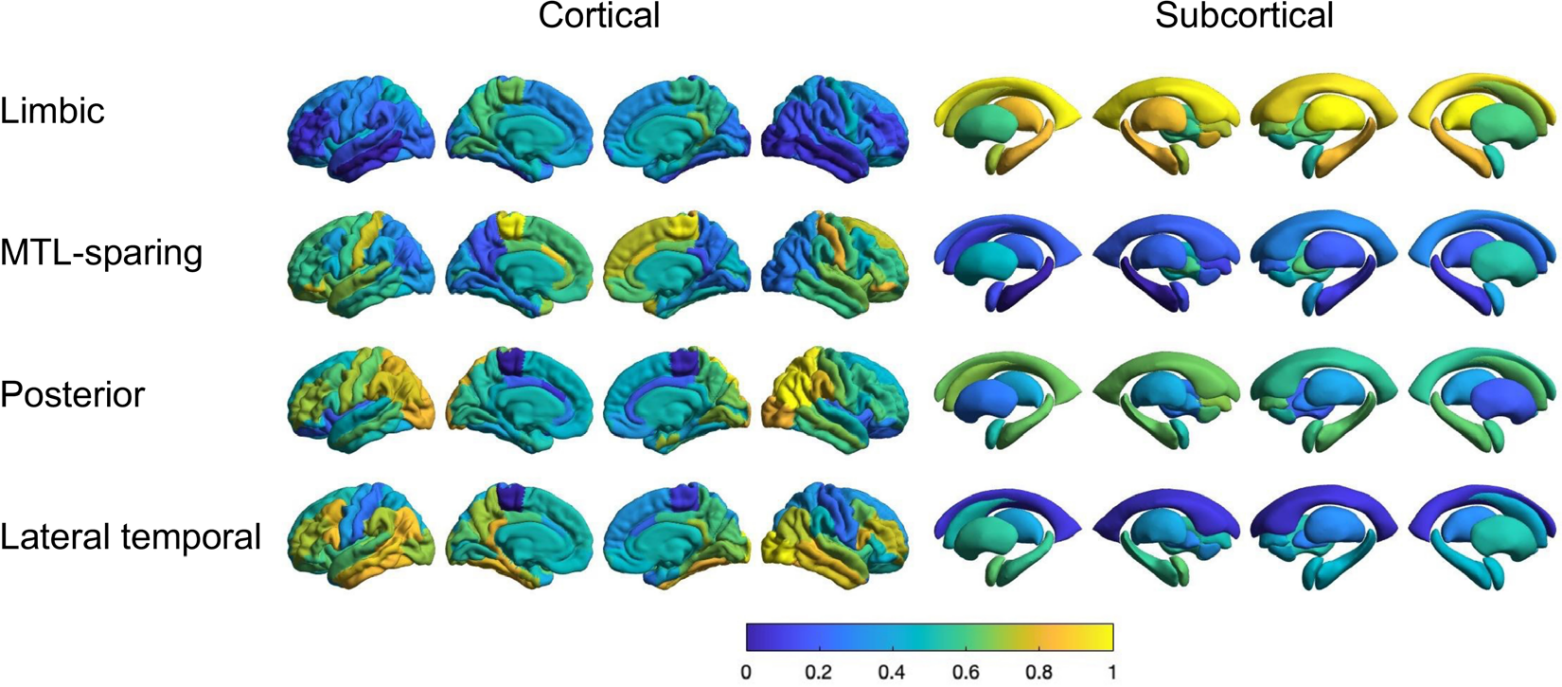
Tau distribution for each subtype identified by SCCA clustering is visualized on brain surface maps. Four subtypes, namely limbic, MTL-sparing, posterior, and lateral temporal, are displayed separately for both cortical (left) and subcortical (right) regions. The color map represents tau deposition levels. The distributions are calculated using the average z-scores of tau PET scans for subjects within each subtype and normalized across all subtypes to a scale of 0 to 1 for visualization.

Figure 4 presents the normalized spatial patterns for each identified tau subtype. Subtype 1 demonstrated tau accumulation predominantly within MTL regions typically affected early in AD. Subtype 2 exhibited a parietal-dominant tau distribution with relative sparing of the MTL. Subtype 3 showed predominant tau deposition in posterior occipitotemporal regions, aligning with previously described posterior-predominant AD subtypes. Subtype 4 displayed a distinct lateral temporal-predominant pattern. The identified spatial patterns align closely with prior imaging-based tau subtyping studies (Vogel et al., 2021), supporting the robustness and clinical relevance of our Elastic SCCA clustering framework. Because individuals within each subtype are at different stages of disease progression, normalization was applied to enable clearer visualization of subtype-specific spatial patterns. Unnormalized SUVR maps are provided in Supplementary Figure S3. Individual tau PET SUVR maps sampled from participants within each subtype are provided in Supplementary Figure S4.

#### 3.1.2 Subtyping with Aβ and genetics

Motivated by previous Aβ PET subtyping studies, we applied Elastic SCCA clustering under a two subtypes setting. The resulting subtypes showed distinct and reproducible regional deposition patterns. To facilitate comparisons across subtypes, average regional Aβ PET z-scores were computed and normalized within each subtype to a 0–1 scale.

**Fig. 5.**
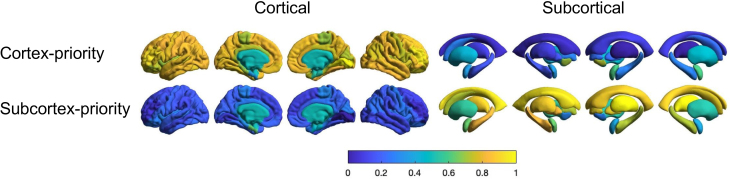
Aβ PET distribution for each subtype identified by SCCA clustering is visualized on brain surface maps. Two subtypes, namely cortex-priority and subcortex-priority, are displayed separately for both cortical (left) and subcortical (right) regions. The color map represents the level of Aβ deposition. The distributions are calculated using the average z-scores of Aβ PET scans for subjects within each subtype and normalized across all subtypes to a scale of 0 to 1 for visualization.

Figure 5 illustrates normalized spatial patterns for the two identified Aβ subtypes. The cortex-priority subtype demonstrated widespread cortical involvement, particularly in the precuneus, posterior cingulate, and frontal cortex, consistent with regions commonly implicated in early amyloid accumulation. The subcortex-priority subtype, in contrast, was characterized by prominent deposition in the basal ganglia and thalamus. While most previous studies have described amyloid progression as beginning in cortical regions with subsequent subcortical involvement (Thal et al., 2002), our findings indicate the presence of a subcortical-predominant variant, consistent with data-driven reports of alternative trajectories (Sun et al., 2023). Together, these findings indicate that while cortical involvement represents the dominant pattern of Aβ deposition, clustering approaches can also reveal less common but distinct spatial variants. Corresponding unnormalized SUVR maps are provided in Supplementary Figure S3, and representative individual maps are shown in Supplementary Figure S5.

#### 3.1.3 Joint distribution of tau and Aβ subtypes

**Fig. 6.**
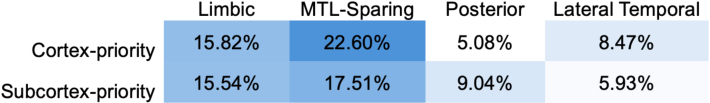
Joint distribution of tau and Aβ subtypes in the same subjects. Percentages indicate the proportion of participants classified into each tau subtype (limbic, MTL-sparing, posterior, lateral temporal) within the two Aβ subtypes (cortex-priority and subcortex-priority). Darker shading reflects a higher proportion of subjects in a given combination. The MTL-sparing subtype shows the highest frequency within both Aβ subtypes, whereas the posterior and lateral temporal subtypes are less common.

Given that tau and Aβ deposition represent distinct but interrelated hallmarks of AD pathology (Abyadeh et al., 2024), we examined their joint distribution in the 354 participants with both PET modalities available. Figure 6 illustrates the overlap between tau and Aβ subtypes. Across both amyloid subtypes, the MTL-sparing tau subtype was most frequent, followed by the limbic subtype, whereas the posterior and lateral temporal tau subtypes were relatively uncommon. Within these less frequent patterns, relative differences emerged. Cortex-priority Aβ cases showed a slightly higher proportion of the lateral temporal tau subtype, whereas subcortex-priority Aβ cases more often overlapped with the posterior tau subtype. These cross-modality associations suggest that amyloid deposition patterns may shape the regional expression of tau pathology, and that the SCCA clustering framework can jointly characterize their shared genetic underpinnings.

### 3.2 Sparse canonical vectors for subtypes

**Fig. 7.**
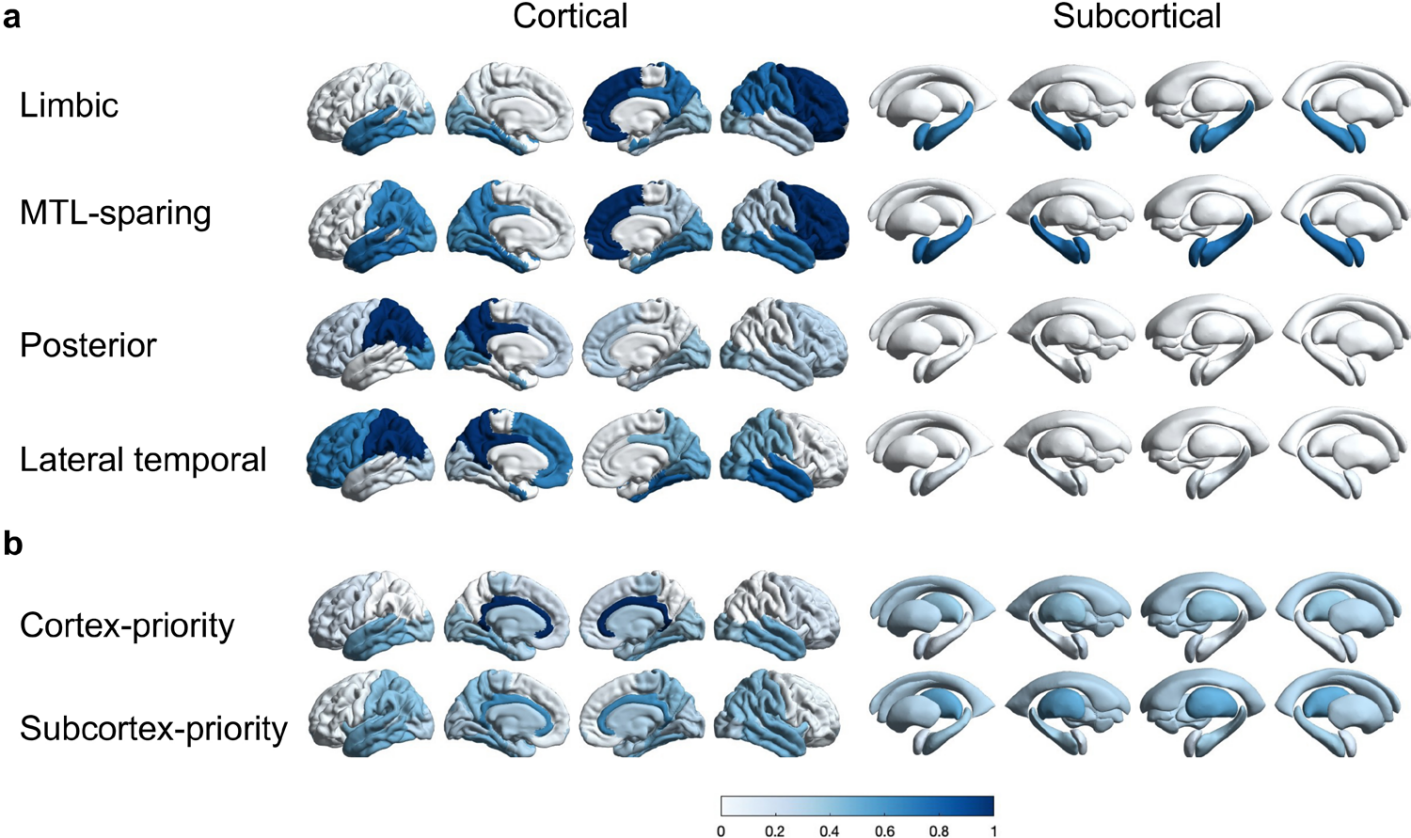
Brain surface plots showing the phenotypic sparse canonical vectors for the tau and Aβ subtypes identified by SCCA clustering. (a) Sparse canonical vectors for the four tau subtypes: limbic, MTL-sparing, posterior, and lateral temporal. (b) Sparse canonical vectors for the two Aβ subtypes: cortex-priority and subcortex-priority. Canonical vectors are presented across cortical and subcortical regions, with the color map indicating the relative weight of each region. Darker blue highlights regions with higher weights, emphasizing their importance for each subtype.

As described in Section 2, each subtype is associated with sparse canonical vectors derived from PET images and genetics data. These sparse canonical vectors reveal which features in each subtype contribute most to the correlations. Figure 7a shows the phenotypic sparse canonical vectors for tau subtypes visualized as brain surface plots. We notice two broad patterns in the vectors: high MTL weights in the limbic and MTL-sparing subtypes and a less pronounced MTL role in the posterior and lateral temporal subtypes. Limbic and MTL-sparing subtypes have similar MTL weights but differ in parietal contributions, which are inversely related. Additionally, the MTL-sparing subtype exhibits higher weights in temporal regions compared to the limbic subtype. For posterior and lateral temporal subtypes, parietal contributions are critical, with the posterior subtype emphasizing occipital regions and the lateral temporal subtype focusing on temporal regions. Figure 7b shows phenotypic sparse canonical vectors for Aβ subtypes. Here, we see balanced contributions from cortical and subcortical regions. The cortex-priority subtype has the highest weights in the cingulate cortex, highlighting its role in cortical Aβ accumulation. In contrast, the subcortex-priority subtype shows stronger contributions from subcortical regions, particularly the basal ganglia and hippocampus.

### 3.3 Genetic signatures of subtypes

**Fig. 8.**
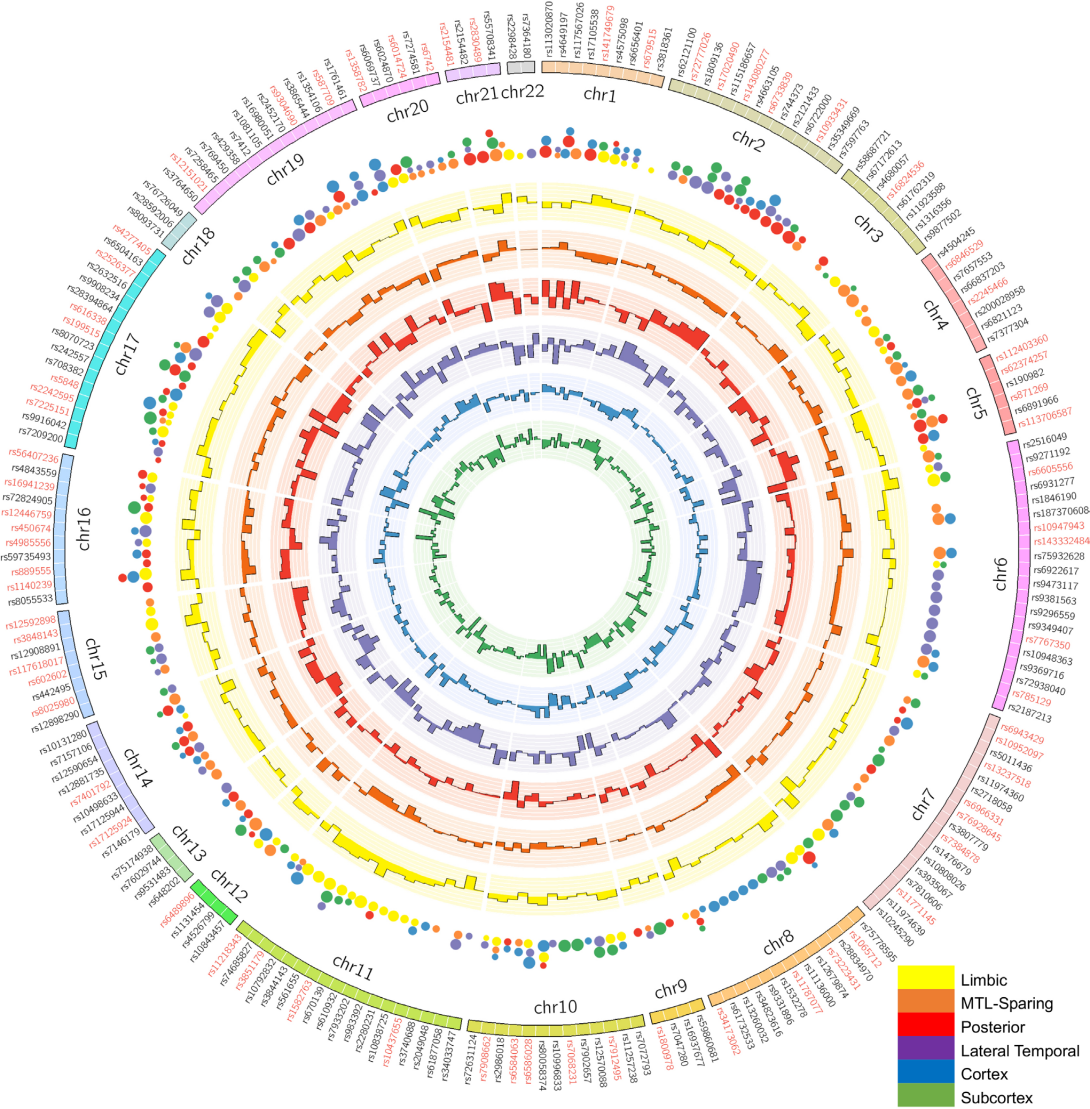
Circular visualization of subtype-specific SNP contributions. The outer ring shows chromosome ideograms. The inner annular histograms depict the mean z-scored SNP dosage for each subtype, plotted at their chromosomal positions. Bar length reflects the absolute magnitude of the dosage, and bar direction indicates its sign (outward = positive, inward = negative). Taller bars, therefore, denote stronger contributions of SNPs to the corresponding subtype. Colored dots outside the histograms mark the top-ranked SNPs (e.g., top 60 per subtype based on dosage rank). Larger dots correspond to higher-ranking SNPs, highlighting the most influential variants for each subtype.

To characterize distinct SNP dosage patterns for each subtype, we averaged the z-scored dosage across subjects within each subtype and visualized the results with a circular plot (Fig. 8). Each color represents a different subtype: tau subtypes include limbic (yellow), MTL-sparing (orange), posterior (red), and lateral temporal (purple), while Aβ subtypes include cortex-priority (blue) and subcortex-priority (green). Bars depict subtype-specific SNP dosage, with bar direction indicating the sign (outward = positive, inward = negative) and bar length indicating magnitude. Positive values show that the SNP dosage in subjects of a given subtype is higher than the cohort mean, while negative values represent a lower dosage. To highlight the most influential variants, the top 60 SNPs by rank in each subtype are plotted as dots on the outer ring, with dot size corresponding to ranking strength (see Supplementary Tables S3–S4 for full lists). The outermost annular ring lists SNPs in chromosomal order, and SNPs annotated as AD risk loci by the Alzheimer’s Disease Sequencing Project Gene Verification Committee are highlighted in red.

The dosage profiles across subtypes revealed both shared and distinct genetic contributions. Several established AD risk loci, including APOE, BIN1, PICALM, and CLU, appeared across multiple subtypes, underscoring their broad involvement in disease susceptibility. At the same time, subtypes exhibited distinct genetic patterns. For example, the limbic tau subtype showed higher dosage of MS4A4E (rs670139), DAPK2 (rs12908891), and HLA-DRB1/DRB5 (rs9271192), while the posterior subtype exhibited elevated dosage of CSMD1, INPP5D, MEF2C, and APP. The lateral temporal subtype was enriched for ABI3 (rs616338), APOE (rs429358), and NCK2. For Aβ subtypes, the cortex-priority group showed higher dosage of variants in HSD17B6/SDR9C7, GRN, SORL1, and SIGLEC11, whereas the subcortex-priority group featured stronger representation of MYO15A, PLCG2, GLIS3, and ANK3. Importantly, APOE ε4 variants (rs429358 and rs7412) were not uniformly enriched across all subtypes. They ranked prominently in the lateral temporal tau and cortex-priority Aβ groups, but were less pronounced in others, suggesting that while APOE remains a major genetic risk factor, additional variants substantially contribute to the genetic architectures of the subtypes.

While many of the top-ranked SNPs correspond to well-established AD loci, others such as MYO15A or DAPK2 are less commonly reported in AD genetics literature. Their presence in the dosage profiles may reflect exploratory signals arising from multivariate associations or cohort-specific variability. These findings should, therefore, be considered hypothesis-generating, requiring replication in larger, independent datasets before firm biological conclusions can be drawn.

### 3.4 Cognitive profiles for subtypes

**Fig. 9.**
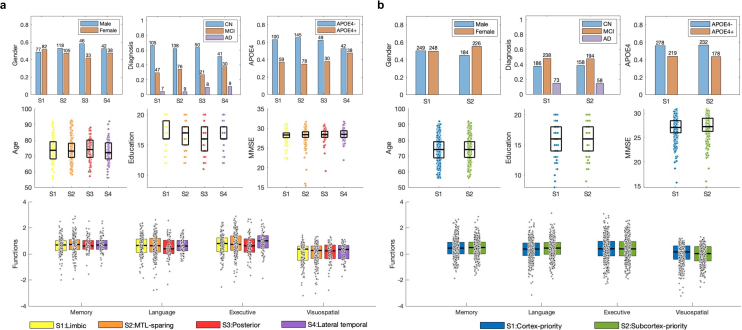
(a) Demographic and cognitive profiles for the four tau subtypes: limbic, MTL-sparing, posterior, and lateral temporal. (b) Demographic and cognitive profiles for the two Aβ subtypes: cortex-priority and subcortex-priority. The plots include age, education, MMSE scores, gender distribution, diagnosis (CN, MCI, AD), APOE4 status, and performance on cognitive functions (memory, language, executive, and visuospatial) across the subtypes. The top row illustrates bar charts for gender, diagnosis, and APOE4 status. The middle row in each panel depicts box plots for age, education, and MMSE scores, while the bottom row shows comparisons of subtypes in cognitive function scores.

We examined the cognitive, demographic, and longitudinal clinical profiles associated with each identified subtype to evaluate their clinical relevance. The four domain-specific cognitive scores (memory, language, executive function, and visuospatial ability) were adjusted for age, sex, clinical diagnosis (CN/MCI/AD), and years of education prior to comparison to reduce potential confounding. Figure 9 summarizes key demographic and cognitive measures, including age, sex, APOE4 status, MMSE, and the adjusted domain-specific scores. Among the tau subtypes, the limbic variant is associated with the most severe memory impairment, consistent with its high tau burden in the medial temporal lobe, a region critical for episodic memory. In contrast, the MTL-sparing subtype shows relatively preserved memory function but exhibits deficits in executive and visuospatial domains, reflecting its greater parietal involvement. It also demonstrates stronger memory performance compared to other functions and is less likely to include APOE4 carriers. The lateral temporal subtype presents with pronounced language deficits but relatively preserved memory performance, in line with tau accumulation in language-related cortices. The posterior subtype shows the poorest performance in both language and executive domains, consistent with its occipital and parietal pathology and aligning with previous reports of posterior cortical atrophy (Li et al., 2018). In the case of Aβ subtypes, individuals classified within the cortex-priority subtype tend to perform worse in global cognition, particularly in memory and executive function, compared to those in the subcortex-priority subtype. The cortex-priority subtype also shows a higher prevalence of APOE4 carriers, suggesting a genetic contribution to Aβ deposition patterns. Supplementary Figure S6 provides a simplified visualization of subtype-specific performance trends across MMSE and the four cognitive domains, which facilitates comparison and highlights differences among subtypes.

**Fig. 10.**
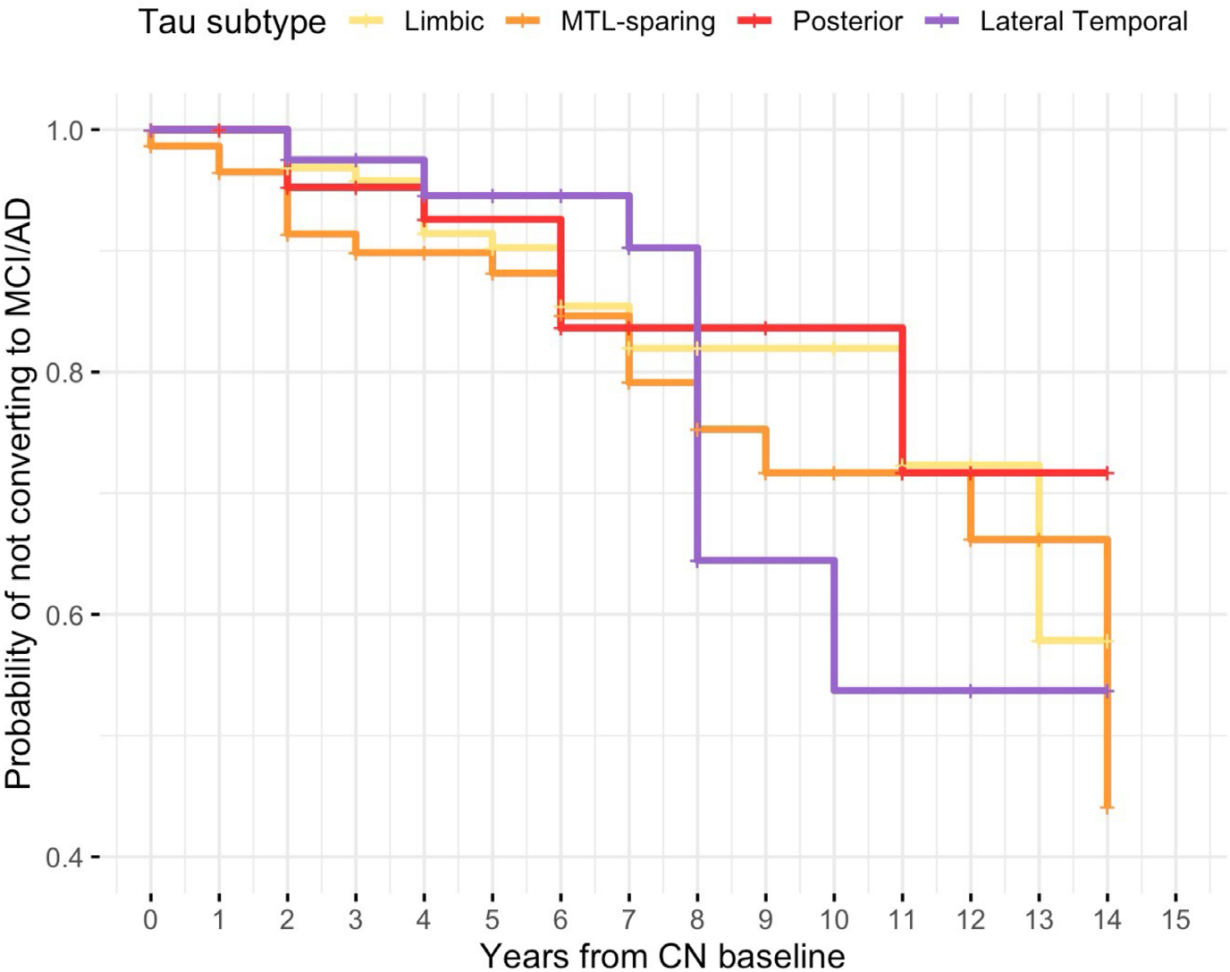
Kaplan-Meier curves showing the probability of individuals in the CN group not converting to MCI or AD based on longitudinal diagnosis, grouped by tau PET subtypes derived from SCCA clustering. Participants were classified into four subtypes: Limbic, MTL-sparing, Posterior, and Lateral temporal, according to regional tau PET SUVR patterns. The y-axis indicates the probability of not converting to MCI/AD, and the x-axis represents years from the CN baseline.

To further assess longitudinal progression, we analyzed diagnostic conversion from CN to MCI or AD using longitudinal clinical diagnoses across visits. At baseline, 345 CN participants were included in the analysis. Of these, 319 had at least one follow-up visit, providing a total of 1,199 intervals between consecutive assessments. The mean inter-visit interval was 1.9 years (s.d. = 1.1; median = 2). Figure 10 shows Kaplan-Meier curves suggesting clear differences in conversion risk across tau subtypes. The posterior subtype demonstrated the slowest rate of progression, while the lateral temporal subtype remained relatively steady over time. These findings indicate that genetically informed tau subtypes are not only associated with distinct cognitive phenotypes but also demonstrate different clinical trajectories, highlighting their prognostic relevance. Notably, the subtype-specific progression patterns observed are consistent with trajectories reported in prior imaging-based subtyping studies (Vogel et al., 2021).

### 3.5 Genetic pathway and cell-type signatures of subtypes

**Fig. 11.**
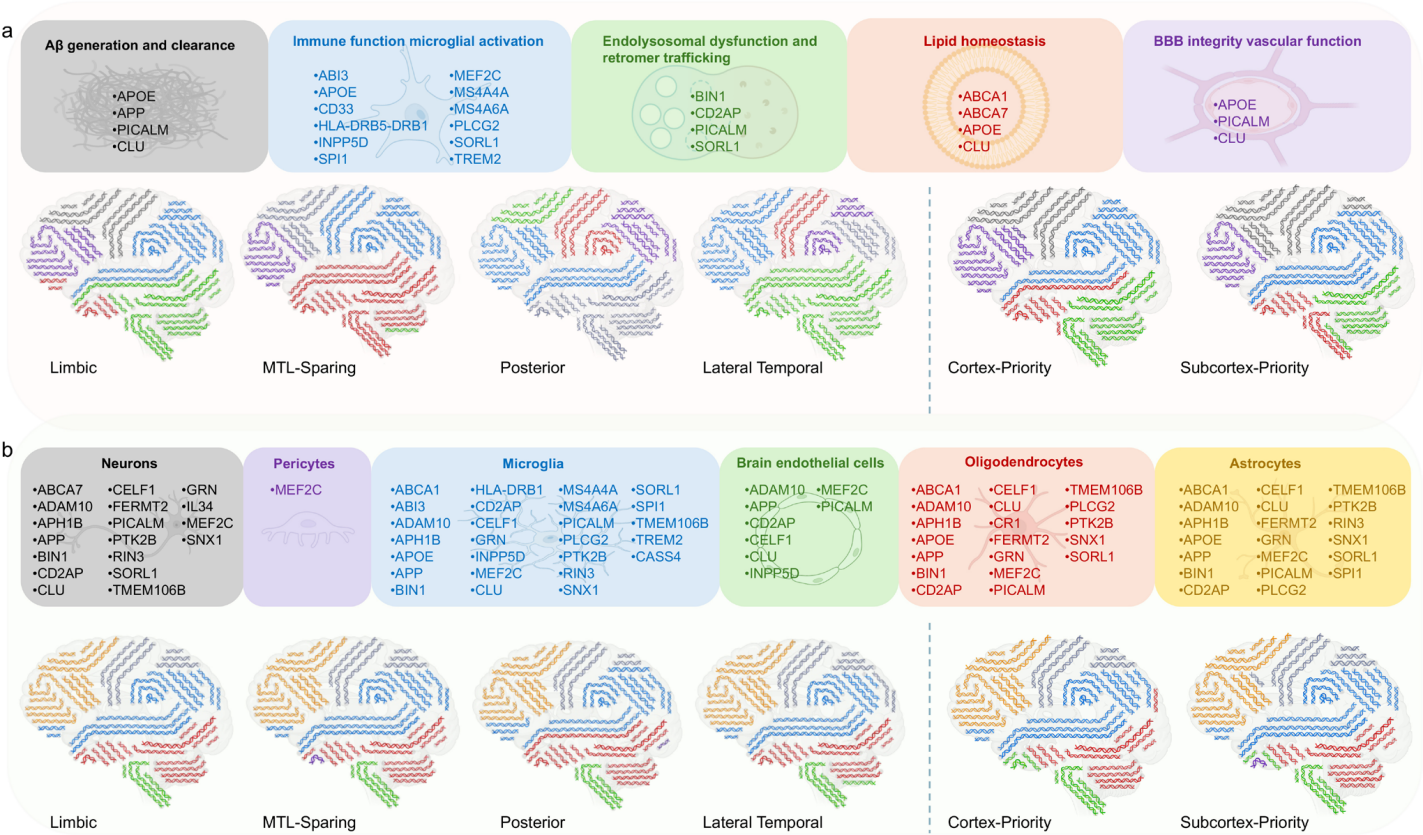
AD genetic risk patterns across tau subtypes (limbic, MTL-sparing, posterior, and lateral temporal) and Aβ subtypes (cortex-priority and subcortex-priority). (a) The proportions of different colors are used to capture the relative proportions of genes corresponding to different biological pathways, including Aβ generation and clearance (grey), immune function and microglial activation (blue), endolysosomal dysfunction and retromer trafficking (green), lipid homeostasis (red), and BBB integrity and vascular function (purple). (b) The proportions of different colors are used to capture the relative proportions of genes corresponding to different cell types, including neurons (grey), astrocytes (yellow), microglia (blue), brain endothelial cells (green), oligodendrocytes (red), and pericytes (purple).

As highlighted in prior studies, genetic risk factors for AD converge on several interconnected biological processes, including amyloid generation and clearance, immune and microglial function, endolysosomal trafficking, lipid metabolism, and blood-brain barrier (BBB) integrity. To link the subtype-associated SNPs within these known mechanisms, we mapped them to established biological pathways and cell-type annotations (gene assignments based on Young-Pearse et al., 2023). Figure 11a shows the proportional representation of the different biological processes and pathways (indicated by different colors) in gene dosages of each tau and Aβ subtype. The lateral temporal tau subtype exhibits the highest concentration of risk genes affecting endolysosomal dysfunction and retromer trafficking. The MTL-sparing tau subtype shows the greatest impact on lipid homeostasis, while the limbic tau subtype shows the least involvement in this domain. The posterior tau subtype presents a more balanced distribution of risk genes across all categories, with a slightly higher involvement in Aβ generation and clearance. The cortex-priority Aβ subtype shows higher involvement of Aβ generation and clearance genes, while the subcortex-priority Aβ subtype demonstrates greater connections to immune function and BBB integrity. Figure 11b illustrates the proportional representation of the different cell types (indicated by different colors) in gene dosages of each tau and Aβ subtype. In contrast to the genetic risk components for biological pathways, the risk components for different cell types are relatively consistent across the tau and Aβ subtypes, suggesting that genetic risk profiles in terms of cell-type-specific expression are not markedly different.

## 4 Discussion

In this work, we developed an SCCA clustering framework for AD subtyping that jointly analyzes PET imaging and genetic data. By integrating tau and Aβ PET with SNP dosage, the method identified biologically and clinically meaningful subtypes characterized by distinct spatial patterns of protein deposition and associated genetic risk signatures. These results demonstrate the feasibility of combining imaging and genetic modalities for subtype discovery and highlight the promise of genetically informed neuroimaging approaches to capturing the heterogeneity of AD.

Our findings align with previous subtyping studies while advancing them in several respects. For tau PET, the four subtypes we identified, namely limbic, MTL-sparing, posterior, and lateral temporal, closely mirror the trajectories reported by Vogel et al. (2021), supporting the reproducibility of our framework. Similarly, for Aβ PET, the cortex-priority and subcortex-priority patterns are consistent with recent ADNI-based work (Sun et al., 2023), although alternative studies such as Collij et al. (2022) have reported three amyloid subtypes in larger datasets. Taken together, these findings illustrate that PET-based subtyping schemes are not uniquely defined, but depend on both analytical choices and cohort characteristics. This underscores the importance of harmonized analyses in larger and more diverse datasets. In this context, our framework extends imaging-based subtyping by incorporating genetic information, enabling PET patterns to be examined along with SNP dosage, and furthermore, at the pathway level. This positions our work alongside recent imaging genetics approaches such as Gene-SGAN (Z. Yang et al., 2024), which leverages deep learning and large-scale MRI data (datasets from 28,858 individuals). Unlike AI-based approaches, our method is based on a mathematical model and hence better suited for data-limited settings.

In addition to the methodological contribution, genetically informed PET subtypes may be relevant for translational and clinical applications. Subtypes defined by imaging-genetics may different in disease progression, cognitive decline, or responsiveness of targeted therapies. For instance, the cortex-priority Aβ subtype showed lower cognitive scores and higher APOE4 prevalence, suggesting prognostic implications. Similarly, tau subtypes displayed domain-specific cognitive profiles, such as memory impairment in the limbic group and language deficits in the lateral temporal group. These distinctions could eventually inform precision medicine strategies, including risk evaluation, refinement of diagnostic criteria, and the design of subtype-specific treatment.

Several biological and data-related limitations should be considered when interpreting our findings. Subcortical Aβ signals are susceptible to off-target binding and nonspecific uptake, particularly in white matter regions, which reduces their biological interpretability and may contribute to the subcortex-priority subtype. Additionally, the analysis did not restrict participants to Aβ-positive individuals. This choice was intended to capture the full disease continuum, but it may also contribute to apparent separations between biomarker-positive and biomarker-negative cases. Given the current sample size, conducting a robust subtype analysis restricted to biomarker-positive individuals was not feasible, and replication in larger cohorts will be important to determine whether the subcortical subtype represents a reproducible and clinically meaningful pattern. From a genetic perspective, AD is highly heritable, yet only a small proportion of the variance is explained by known SNPs, and uncertainty remains in assigning effector genes and biological pathways for many variants. A more complete characterization of the genetic architecture of AD will likely improve the performance of SCCA clustering in discerning subtypes. Finally, the dataset used in this study was derived entirely from ADNI, with a modest number of tau PET scans, which limits generalizability and statistical power; validation in independent cohorts with broader demographic and biological diversity will therefore be crucial.

Beyond data-related constraints, several methodological limitations should also be considered. First, the alternating optimization underlying SCCA clustering is non-convex, meaning that solutions may depend on initialization and may not achieve a global optimum. Although we mitigated this by perturbing the initial vectors and performing bootstrap resampling, future work with larger datasets and external replication will be important for establishing population-level stability. Second, the linear assumption of CCA may underestimate nonlinear genotype–phenotype relationships; extensions such as kernel or deep CCA may better capture complex interactions. Third, while our descriptive analyses highlight clear subtype-specific patterns, we did not perform extensive statistical testing across subtypes because stage heterogeneity within each subtype may confound such comparisons. Finally, although this study predefined four tau and two Aβ clusters to align with prior PET-based subtyping literature, applications of the SCCA-clustering framework to novel cohorts may require data-dependent determination of the number of clusters. In exploratory settings, users may evaluate a range of candidate K values (e.g., K= 2 – 6) and select K by jointly considering the convergence of the cost function defined in Equation 14 or 18, stability under bootstrap resampling or repeated initializations, separation of canonical scores, and the interpretability and adequate sample size of each resulting subtype.

## 5 Conclusion

We presented a novel SCCA clustering framework for AD subtyping that integrates PET imaging with genetic information. This approach identified tau and Aβ subtypes with distinct spatial and genetic signatures consistent with previous imaging-only subtyping efforts. These findings demonstrate the potential of genetically informed PET subtyping to improve our understanding of AD heterogeneity. As future work, we plan to validate the framework in larger and more varied cohorts. We also plan to explore the incorporation of additional biomarkers and broader forms of genetic data to refine subtype definition and enhance biological interpretability.

## Ethics

This study used data obtained from the ADNI database. The ADNI study was approved by the Institutional Review Boards (IRBs) of all participating institutions, and written informed consent was obtained from all participants. No new data were collected for this study, and all procedures were performed in accordance with the ethical standards of the Declaration of Helsinki.

## Supplementary Material

Supplementary Material

## Data Availability

The data used in this study are sourced from the ADNI, a publicly available database. Due to privacy and data-sharing agreements, the raw data are not included in this repository. Researchers interested in accessing the data can obtain it directly from the ADNI website, following the required data request procedures. The code is available at https://www.github.com/bidslabumass/SCCA-Clustering
