# Supplementary material for: Unveiling unified patterns in Alzheimer’s disease subtypes: An SCCA clustering approach integrating PET imaging and genomics data
Source: Imaging Neurosci (Camb). 2026 Feb 25;4:IMAG.a.1151. doi: 10.1162/IMAG.a.1151 (PMC12938741; doi:10.1162/IMAG.a.1151)
Supplement: Supplementary Material [file IMAG.a.1151_supp.pdf]

Supplementary Material

Supplement Table S1: Tau spatial features and corresponding brain regions.

Spatial features	Regions
MTL	Entorhinal<br>Hippocampus<br>Amygdala
Temporal	Parahippocampal<br>Inferior temporal<br>Fusiform<br>Middle temporal<br>Superior temporal<br>Transverse temporal
Parietal	Isthmus cingulate<br>Inferior parietal<br>Precuneus<br>Superior parietal<br>Supramarginal<br>Posterior cingulate<br>Postcentral
Occipital	Lateral occipital<br>Lingual<br>Pericalcarine<br>Cuneus
Frontal	Caudal anterior cingulate<br>Caudal middle frontal<br>Lateral orbitofrontal<br>Medial orbitofrontal<br>Pars opercularis<br>Pars orbitalis<br>Pars triangularis<br>Precentral<br>Rostral anterior cingulate<br>Rostral middle frontal<br>Superior frontal<br>Insula

Supplement Table S2: List of SNPs and reference.

Chr.	Position	A1	A2	Risk allele	Gene	SNPs	Reference
1	985377	C	T	T	AGRN	rs113020870	Wightman et al. (2021)
1	24495722	G	A	G	IFNLR1	rs4649197	Kondo et al. (2022)

Chr.	Position	A1	A2	Risk allele	Gene	SNPs	Reference
1	58715824	C	G	C	DAB1	rs117567026	Kondo et al. (2022)
1	81315043	A	G	G	ELTD1,LPHN2	rs17105538	Yan et al. (2021)
1	109888432	T	C	C	SORT1	rs141749679	Bellenguez et al. (2022)
1	161155392	G	A	A	ADAMTS4	rs4575098	Jansen et al. (2020)
1	207692049	A	G	A	CR1	rs6656401	Van Cauwenberghe et al. (2016)
1	207750568	T	C	T	CR1	rs679515	Wightman et al. (2021)
1	207784968	A	G	A	CR1	rs3818361	"Alzgene Website" (n.d.)
2	3093952	T	G	T	LINC01250	rs62121100	Yan et al. (2021)
2	9699011	A	G	G	ADAM17	rs72777026	Bellenguez et al. (2022)
2	11152180	G	C	C	KCNF1,FLJ33534	rs1809136	Yan et al. (2021)
2	37531939	T	C	C	PRKD3	rs17020490	Bellenguez et al. (2022)
2	106235428	A	C	C	NCK2	rs115186657	Wightman et al. (2021)
2	106366056	T	C	C	NCK2	rs143080277	Bellenguez et al. (2022)
2	127891427	A	C	C	BIN1	rs4663105	Wightman et al. (2021)
2	127892810	C	T	T	BIN1	rs6733839	Van Cauwenberghe et al. (2016)
2	127894615	A	G	G	BIN1	rs744373	Franzmeier et al. (2019)
2	149557860	T	C	C	EPC2	rs2121433	S. Kim et al. (2011)
2	209075957	A	G	G	C2orf80,IDH1	rs6722000	Yan et al. (2021)
2	233981912	G	C	C	INPP5D	rs10933431	Jansen et al. (2020)
2	234068476	C	T	T	INPP5D	rs35349669	Van Cauwenberghe et al. (2016)
2	234082577	A	C	C	INPP5D	rs7597763	Wightman et al. (2021)
3	65873820	T	G	T	MAGI1	rs58687721	Kondo et al. (2022)
3	77035984	T	C	T	ROBO2	rs67172613	Kondo et al. (2022)
3	153096985	G	A	A	RAP2B,C3orf79	rs4680057	Yan et al. (2021)
3	154787511	G	A	G	MME	rs16824536	Bellenguez et al. (2022)
3	154801978	A	G	G	MME	rs61762319	Bellenguez et al. (2022)
3	184459667	C	T	C	MAGEF1,LOC101928992	rs11923588	Yan et al. (2021)
3	190657163	T	C	C	SNAR-I	rs1316356	Cruchaga et al. (2013)
3	190669518	G	A	A	GMNC	rs9877502	Jansen et al. (2022)
4	11014822	A	G	A	CLNK	rs4504245	Wightman et al. (2021)
4	11025131	C	T	C	CLNK	rs6846529	Bellenguez et al. (2022)
4	11723235	A	G	A	HS3ST1	rs7657553	Jansen et al. (2020)
4	36897136	C	T	T	DTHD1,MIR4801	rs66837203	Yan et al. (2021)
4	40198846	G	C	G	RHOH	rs2245466	Bellenguez et al. (2022)
4	70923661	G	A	G	HTN1	rs200028958	Yan et al. (2021)
4	122249973	T	C	C	QRFPR	rs6821123	Kondo et al. (2022)
4	187129780	T	G	T	CYP4V2	rs7377304	Yan et al. (2021)
5	14724413	T	A	A	ANKH	rs112403360	Bellenguez et al. (2022)
5	86223195	T	C	C	COX7C	rs62374257	Bellenguez et al. (2022)
5	88223420	G	A	A	MEF2C	rs190982	Van Cauwenberghe et al. (2016)
5	150432388	C	T	C	TNIP1	rs871269	Wightman et al. (2021)

Chr.	Position	A1	A2	Risk allele	Gene	SNPs	Reference
5	156526331	A	G	G	HAVCR2	rs6891966	Wightman et al. (2021)
5	179628150	G	A	A	RASGEF1C	rs113706587	Bellenguez et al. (2022)
6	32570400	T	C	T	HLA-DRB1	rs2516049	Yokoyama et al. (2016)
6	32578530	C	A	C	HLA-DRB1/HLA-DRB5	rs9271192	Van Cauwenberghe et al. (2016)
6	32583099	A	G	A	HLA-DQA1	rs6605556	Bellenguez et al. (2022)
6	32583357	A	T	A	HLA-DRB1	rs6931277	Jansen et al. (2020)
6	32583813	G	A	G	HLA-DRB1	rs1846190	Wightman et al. (2021)
6	40942196	G	A	A	TREM2	rs187370608	Wightman et al. (2021)
6	41004093	G	A	G	UNC5CL	rs10947943	Bellenguez et al. (2022)
6	41129207	C	T	T	TREM2	rs143332484	Bellenguez et al. (2022)
6	41129252	C	T	T	TREM2	rs75932628	Guerreiro et al. (2013)
6	41336101	G	A	G	NCR2	rs6922617	Cruchaga et al. (2013)
6	47431284	A	C	C	CD2AP	rs9473117	Naj et al. (2011)
6	47432637	C	T	C	CD2AP	rs9381563	Jansen et al. (2020)
6	47452270	T	C	C	CD2AP	rs9296559	Xue et al. (2022)
6	47453378	G	C	C	CD2AP	rs9349407	"Alzgene Website" (n.d.)
6	47485126	C	T	T	CD2AP	rs7767350	Bellenguez et al. (2022)
6	47487762	A	G	G	CD2AP	rs10948363	Van Cauwenberghe et al. (2016)
6	47552180	A	T	T	CD2AP	rs9369716	Wightman et al. (2021)
6	105721926	A	G	A	PREP	rs72938040	Kondo et al. (2022)
6	114612895	T	C	T	HS3ST5	rs785129	Bellenguez et al. (2022)
6	162634337	G	A	G	PARK2	rs2187213	Chen et al. (2017)
7	7856894	T	C	T	UMAD1	rs6943429	Bellenguez et al. (2022)
7	8244012	T	C	T	ICA1	rs10952097	Bellenguez et al. (2022)
7	12268758	A	C	A	TMEM106B	rs5011436	Wightman et al. (2021)
7	12269593	C	A	C	TMEM106B	rs13237518	Bellenguez et al. (2022)
7	30370786	A	G	A	ZNRF2	rs11974360	Kondo et al. (2022)
7	37841534	A	G	A	NME8	rs2718058	Van Cauwenberghe et al. (2016)
7	37883793	T	C	C	EPDR1	rs6966331	Bellenguez et al. (2022)
7	54941328	C	T	C	SEC61G	rs76928645	Bellenguez et al. (2022)
7	77671455	C	T	C	MAGI2	rs3807779	H. R. Kim et al. (2021)
7	99932049	C	T	T	ZCWPW1/NYAP1	rs7384878	Wightman et al. (2021)
7	100004446	C	T	T	ZCWPW1	rs1476679	Van Cauwenberghe et al. (2016)
7	143099133	C	A	C	EPHA1	rs10808026	Kunkle et al. (2019)
7	143104331	G	C	C	EPHA1-AS1	rs3935067	Wightman et al. (2021)
7	143108158	T	C	C	EPHA1	rs7810606	Jansen et al. (2020)
7	143110762	G	A	G	EPHA1	rs11771145	Van Cauwenberghe et al. (2016)
7	148438804	C	T	T	CUL1	rs11974639	Kondo et al. (2022)
7	148530294	T	C	C	EZH2	rs10245290	Kondo et al. (2022)
8	4801168	G	A	A	CSMD1	rs75778595	Kondo et al. (2022)
8	11702122	G	C	C	CTSB	rs1065712	Bellenguez et al. (2022)

Chr.	Position	A1	A2	Risk allele	Gene	SNPs	Reference
8	27195121	T	C	C	PTK2B	rs28834970	Van Cauwenberghe et al. (2016)
8	27219987	C	T	T	PTK2B	rs73223431	Kunkle et al. (2019)
8	27230819	A	G	G	PTK2B	rs12679874	Yokoyama et al. (2016)
8	27464519	T	C	C	CLU	rs11136000	"Alzgene Website" (n.d.)
8	27465312	T	C	C	CLU	rs11787077	Bellenguez et al. (2022)
8	27466315	T	C	C	CLU	rs1532278	Wightman et al. (2021)
8	27467686	C	T	T	CLU	rs9331896	Van Cauwenberghe et al. (2016)
8	106566606	T	C	T	ZFPM2	rs34823616	Kondo et al. (2022)
8	132451455	A	C	C	ADCY8,EFR3A	rs13260032	Yan et al. (2021)
8	145108151	G	A	A	SHARPIN	rs61732533	Wightman et al. (2021)
8	145158607	G	A	A	SHARPIN	rs34173062	Bellenguez et al. (2022)
9	3927804	C	T	T	GLIS3	rs59860681	Cruchaga et al. (2013)
9	19642563	G	A	G	SLC24A2	rs16937677	Kondo et al. (2022)
9	23297808	C	T	C	ELAVL2	rs7047280	Chen et al. (2017)
9	107665978	C	G	G	ABCA1	rs1800978	Bellenguez et al. (2022)
10	6106266	T	C	C	IL2RA	rs7072793	Chen et al. (2017)
10	11717397	T	C	C	ECHDC3	rs11257238	Jansen et al. (2020)
10	11718713	A	G	G	USP6NL/ECHDC3	rs7912495	Wightman et al. (2021)
10	59938336	A	G	A	IPMK	rs12570088	Yokoyama et al. (2016)
10	61738152	G	T	T	CCDC6	rs7902657	Wightman et al. (2021)
10	61784928	T	G	G	ANK3	rs7068231	Bellenguez et al. (2022)
10	67784976	A	G	A	CTNNA3	rs10996833	Kondo et al. (2022)
10	78859025	T	C	T	KCNMA1	rs80058374	Kondo et al. (2022)
10	82253984	C	T	T	TSPAN14	rs6586028	Bellenguez et al. (2022)
10	98026407	G	A	A	BLNK	rs6584063	Bellenguez et al. (2022)
10	105218359	T	C	T	CALHM1	rs2986018	Vacher et al. (2019)
10	124172912	A	G	A	PLEKHA1	rs7908662	Bellenguez et al. (2022)
10	125679317	G	A	G	CPXM2	rs72631124	Kondo et al. (2022)
11	8853774	C	T	C	DENND2B (ST5)	rs34033747	Kondo et al. (2022)
11	26600213	G	A	G	ANO3	rs61877058	Kondo et al. (2022)
11	27750586	G	A	G	AC103796.1-BDNF	rs2049048	Vacher et al. (2019)
11	47380340	G	T	T	MADD/SPI1	rs3740688	Wightman et al. (2021)
11	47391948	G	A	A	SPI1	rs10437655	Bellenguez et al. (2022)
11	47557871	T	C	C	CELF1	rs10838725	Van Cauwenberghe et al. (2016)
11	47600438	C	T	T	DKFZp586K0821/NDUFS3	rs2280231	Yokoyama et al. (2016)
11	59923508	A	G	A	MS4A6A	rs983392	Van Cauwenberghe et al. (2016)
11	59936926	A	C	A	MS4A2	rs7933202	Kunkle et al. (2019)
11	59939307	T	G	G	MS4A6A	rs610932	"Alzgene Website" (n.d.)
11	59971795	G	T	T	MS4A4E	rs670139	"Alzgene Website" (n.d.)
11	60021948	G	A	G	MS4A4A	rs1582763	Wightman et al. (2021)
11	85800279	G	A	A	PICALM	rs561655	Wightman et al. (2021)

Chr.	Position	A1	A2	Risk allele	Gene	SNPs	Reference
11	85850243	C	T	T	PICALM	rs3844143	Jansen et al. (2020)
11	85867875	A	G	G	PICALM	rs10792832	Van Cauwenberghe et al. (2016)
11	85868640	T	C	C	PICALM	rs3851179	"Alzgene Website" (n.d.)
11	121353077	T	G	G	SORL1	rs74685827	Bellenguez et al. (2022)
11	121435587	T	C	T	SORL1	rs11218343	Wightman et al. (2021)
12	29790399	T	C	C	TMTC1	rs10843457	Kondo et al. (2022)
12	57280586	C	T	C	HSD17B6,SDR9C7	rs4526799	Yan et al. (2021)
12	113348870	G	A	A	OAS1	rs1131454	Magusali et al. (2021)
12	113719788	T	C	C	TPCN1	rs6489896	Bellenguez et al. (2022)
13	33635463	T	C	T	KL	rs648202	Vacher et al. (2019)
13	84244873	A	C	A	SLITRK1	rs9531483	Yan et al. (2021)
13	103486018	A	T	A	BIVM-ERCC5	rs76029744	Kondo et al. (2022)
13	108015726	G	A	G	FAM155A	rs75174938	Kondo et al. (2022)
14	53298853	A	G	A	FERMT2	rs7146179	Wightman et al. (2021)
14	53391680	A	G	G	FERMT2	rs17125924	Bellenguez et al. (2022)
14	53400629	T	C	C	FERMT2	rs17125944	Van Cauwenberghe et al. (2016)
14	92926952	G	T	G	SLC24A4/BIN3	rs10498633	Van Cauwenberghe et al. (2016)
14	92931261	G	A	G	SLC24A4	rs7401792	Bellenguez et al. (2022)
14	92932828	T	C	T	SLC24A4	rs12881735	Kunkle et al. (2019)
14	92938855	G	A	G	RIN3	rs12590654	Wightman et al. (2021)
14	106228095	A	G	A	IGH	rs7157106	Bellenguez et al. (2022)
14	107121607	G	A	G	IGH	rs10131280	Bellenguez et al. (2022)
15	31294343	A	T	T	TRPM1	rs12898290	Kondo et al. (2022)
15	50994011	A	G	A	SPPL2A	rs8025980	Bellenguez et al. (2022)
15	59022615	T	C	T	ADAM10	rs442495	Jansen et al. (2020)
15	59057023	T	A	T	ADAM10	rs602602	Wightman et al. (2021)
15	63569902	C	T	T	APH1B	rs117618017	Wightman et al. (2021)
15	64236441	G	A	A	DAPK2	rs12908891	Yan et al. (2021)
15	64423506	G	A	G	SNX1	rs3848143	Bellenguez et al. (2022)
15	79229199	A	G	G	CTSH	rs12592898	Bellenguez et al. (2022)
16	11042239	G	A	A	CLEC16A	rs8055533	Yokoyama et al. (2016)
16	30021402	C	T	C	DOC2A	rs1140239	Bellenguez et al. (2022)
16	31122571	C	T	C	BCKDK	rs889555	Bellenguez et al. (2022)
16	31133100	G	A	G	KAT8	rs59735493	Jansen et al. (2020)
16	70694000	C	A	A	IL34	rs4985556	Bellenguez et al. (2022)
16	79608408	T	C	T	MAF	rs450674	Bellenguez et al. (2022)
16	81773003	G	A	A	PLCG2	rs12446759	Bellenguez et al. (2022)
16	81942028	C	G	C	PLCG2	rs72824905	Sims et al. (2017)
16	86454210	T	A	A	FOXF1	rs16941239	Bellenguez et al. (2022)
16	87225431	G	A	A	C16orf95	rs4843559	Jansen et al. (2022)
16	90170095	G	A	A	PRDM7	rs56407236	Bellenguez et al. (2022)

Chr.	Position	A1	A2	Risk allele	Gene	SNPs	Reference
17	4969940	C	T	T	SCIMP/RABEP1	rs7209200	Wightman et al. (2021)
17	4984447	A	G	A	SCIMP	rs9916042	Jansen et al. (2020)
17	5137047	G	A	A	SCIMP	rs7225151	Bellenguez et al. (2022)
17	18059454	G	A	G	MYO15A	rs2242595	Bellenguez et al. (2022)
17	42430244	C	T	T	GRN	rs5848	Bellenguez et al. (2022)
17	42442344	T	C	C	GRN	rs708382	Wightman et al. (2021)
17	44019712	G	A	A	MAPT	rs242557	Chen et al. (2017)
17	44081064	A	G	A	MAPT	rs8070723	Allen et al. (2014)
17	44856641	G	C	C	WNT3	rs199515	Bellenguez et al. (2022)
17	47297297	T	C	T	ABI3	rs616338	Bellenguez et al. (2022)
17	47450775	G	A	A	ABI3	rs28394864	Wightman et al. (2021)
17	47577348	A	G	A	NGFR	rs9908234	Vacher et al. (2019)
17	56409089	G	C	G	TSPOAP1-AS1	rs2632516	Wightman et al. (2021)
17	56410041	A	G	A	TSPOAP1	rs2526377	Bellenguez et al. (2022)
17	61545779	C	T	T	ACE	rs6504163	Wightman et al. (2021)
17	61548918	C	T	T	ACE	rs4277405	Bellenguez et al. (2022)
18	29088958	C	T	C	SUZ12P1	rs8093731	Jansen et al. (2020)
18	50295649	C	G	G	DCC	rs28592006	Kondo et al. (2022)
18	56189459	T	C	C	ALPK2	rs76726049	Jansen et al. (2020)
19	1046520	T	G	G	ABCA7	rs3764650	Vacher et al. (2019)
19	1050874	A	G	A	ABCA7	rs12151021	Wightman et al. (2021)
19	18533642	T	C	C	SSBP4	rs7258465	Yokoyama et al. (2016)
19	45410444	G	A	G	APOE	rs769450	Du et al. (2020)
19	45411941	T	C	C	APOE	rs429358	Yan et al. (2021)
19	45412079	C	T	C	APOE	rs7412	Genin et al. (2011)
19	45412955	A	C	C	APOE	rs1081105	Du et al. (2020)
19	46345886	T	C	T	SYMPK	rs16980051	Yokoyama et al. (2016)
19	49213504	G	A	A	NTN5	rs2452170	Wightman et al. (2021)
19	50453317	C	T	T	SIGLEC11	rs9304690	Bellenguez et al. (2022)
19	51727962	C	A	C	CD33	rs3865444	Jansen et al. (2020)
19	51737991	T	G	T	CD33	rs1354106	Wightman et al. (2021)
19	54771451	T	C	C	LILRB2	rs587709	Bellenguez et al. (2022)
19	54825174	A	C	C	LILRB2	rs1761461	Wightman et al. (2021)
20	393978	A	G	G	RBCK1	rs1358782	Bellenguez et al. (2022)
20	54995699	C	T	C	CASS4	rs6069737	Wightman et al. (2021)
20	54997568	G	A	G	CASS4	rs6024870	Kunkle et al. (2019)
20	54998544	A	G	A	CASS4	rs6014724	Jansen et al. (2020)
20	55018260	T	C	T	CASS4	rs7274581	Van Cauwenberghe et al. (2016)
20	62374441	T	C	C	SLC2A4RG	rs6742	Bellenguez et al. (2022)
21	27473875	C	T	T	APP	rs2154481	Bellenguez et al. (2022)
21	27520931	G	T	G	APP	rs2154482	Wightman et al. (2021)

Chr.	Position	A1	A2	Risk allele	Gene	SNPs	Reference
21	28148191	C	T	C	ADAMTS1	rs2830489	Bellenguez et al. (2022)
21	45627581	A	T	T	C21orf33,ICOSLG	rs55708341	Yan et al. (2021)
22	21982892	C	T	C	YDJC	rs2298428	Yokoyama et al. (2016)
22	42218856	A	G	A	CCDC134	rs7364180	S. Kim et al. (2011)

Supplementary Table S3a. Top 60 SNPs (average z-scored dosage) for tau—Limbic and MTL-sparing.

No.	Limbic			MTL-sparing		
	Gene	SNP	Z	Gene	SNP	Z
1	MS4A4E	rs670139	0.1746	TPCN1	rs6489896	0.1346
2	DAPK2	rs12908891	0.1612	GMNC	rs9877502	0.1305
3	HLA-DRB1/HLA-DRB5	rs9271192	0.1505	QRFPR	rs6821123	0.1262
4	DOC2A	rs1140239	0.1446	ADAMTS1	rs2830489	0.1163
5	CUL1	rs11974639	0.1370	TREM2	rs75932628	0.1127
6	PLCG2	rs12446759	0.1305	RASGEF1C	rs113706587	0.1121
7	CTSB	rs1065712	0.1295	SNAR-I	rs1316356	0.1064
8	PICALM	rs3844143	0.1253	PREP	rs72938040	0.1010
9	PICALM	rs3851179	0.1233	TREM2	rs187370608	0.0977
10	PICALM	rs10792832	0.1144	TNIP1	rs871269	0.0939
11	ACE	rs6504163	0.1126	HSD17B6,SDR9C7	rs4526799	0.0897
12	ADAMTS4	rs4575098	0.1091	EPHA1	rs11771145	0.0878
13	ACE	rs4277405	0.1063	APOE	rs1081105	0.0878
14	HS3ST1	rs7657553	0.1056	IGH	rs7157106	0.0859
15	SCIMP	rs7225151	0.1034	ANKH	rs112403360	0.0850
16	C21orf33,ICOSLG	rs55708341	0.1031	DAB1	rs117567026	0.0782
17	MS4A6A	rs983392	0.1027	ABI3	rs28394864	0.0773
18	LILRB2	rs1761461	0.1017	FERMT2	rs7146179	0.0767
19	PICALM	rs561655	0.0999	NME8	rs2718058	0.0757
20	MAGEF1,LOC101928992	rs11923588	0.0934	ABCA7	rs3764650	0.0755
21	CSMD1	rs75778595	0.0905	CYP4V2	rs7377304	0.0754
22	CR1	rs6656401	0.0896	SLITRK1	rs9531483	0.0744
23	KCNMA1	rs80058374	0.0861	CLNK	rs6846529	0.0719
24	C16orf95	rs4843559	0.0853	SLC24A4	rs7401792	0.0689
25	RBCK1	rs1358782	0.0838	SLC2A4RG	rs6742	0.0609
26	ECHDC3	rs11257238	0.0820	APH1B	rs117618017	0.0601
27	MS4A6A	rs610932	0.0791	FERMT2	rs17125924	0.0586
28	SNX1	rs3848143	0.0787	SHARPIN	rs61732533	0.0580
29	ALPK2	rs76726049	0.0778	HLA-DRB1	rs1846190	0.0565
30	OAS1	rs1131454	0.0777	NTN5	rs2452170	0.0556
31	IL2RA	rs7072793	0.0753	MAGI1	rs58687721	0.0552
32	PLEKHA1	rs7908662	0.0733	SPPL2A	rs8025980	0.0526
33	GRN	rs708382	0.0723	DTHD1,MIR4801	rs66837203	0.0510
34	MS4A2	rs7933202	0.0720	PRKD3	rs17020490	0.0493
35	TSPAN14	rs6586028	0.0716	ZCWPW1/NYAP1	rs7384878	0.0491
36	DKFZp586K0821/NDUFS3	rs2280231	0.0712	SUZ12P1	rs8093731	0.0489
37	IL34	rs4985556	0.0699	ANO3	rs61877058	0.0472
38	SORL1	rs11218343	0.0698	FERMT2	rs17125944	0.0460

No.	Limbic			MTL-sparing		
	Gene	SNP	Z	Gene	SNP	Z
39	HLA-DRB1	rs2516049	0.0688	CTSH	rs12592898	0.0435
40	MS4A4A	rs1582763	0.0678	CASS4	rs7274581	0.0430
41	HSD17B6,SDR9C7	rs4526799	0.0671	HTN1	rs200028958	0.0430
42	PARK2	rs2187213	0.0667	ZCWPW1	rs1476679	0.0415
43	SYMPK	rs16980051	0.0663	BLNK	rs6584063	0.0379
44	CELF1	rs10838725	0.0654	SIGLEC11	rs9304690	0.0360
45	RHOH	rs2245466	0.0651	ICA1	rs10952097	0.0343
46	GRN	rs5848	0.0638	CASS4	rs6069737	0.0333
47	CLNK	rs4504245	0.0638	MADD/SPI1	rs3740688	0.0333
48	RASGEF1C	rs113706587	0.0629	CASS4	rs6024870	0.0333
49	FOXF1	rs16941239	0.0623	PLEKHA1	rs7908662	0.0310
50	NCR2	rs6922617	0.0609	CLNK	rs4504245	0.0306
51	TSPOAP1-AS1	rs2632516	0.0596	SORL1	rs74685827	0.0304
52	SHARPIN	rs34173062	0.0589	MEF2C	rs190982	0.0293
53	TSPOAP1	rs2526377	0.0585	MYO15A	rs2242595	0.0292
54	CR1	rs3818361	0.0580	MME	rs16824536	0.0285
55	YDJC	rs2298428	0.0579	C21orf33,ICOSLG	rs55708341	0.0274
56	CR1	rs679515	0.0576	COX7C	rs62374257	0.0268
57	MYO15A	rs2242595	0.0571	ELAVL2	rs7047280	0.0254
58	RIN3	rs12590654	0.0544	ABCA7	rs12151021	0.0253
59	CPXM2	rs72631124	0.0523	IFNLR1	rs4649197	0.0236
60	LINC01250	rs62121100	0.0516	CASS4	rs6014724	0.0227

Supplementary Table S3b. Top 60 SNPs (average z-scored dosage) for tau—Posterior and Lateral temporal.

No.	Posterior			Lateral temporal		
	Gene	SNP	Z	Gene	SNP	Z
1	CSMD1	rs75778595	0.2309	ABI3	rs616338	0.2862
2	INPP5D	rs7597763	0.2217	NCK2	rs115186657	0.2749
3	MEF2C	rs190982	0.2107	APOE	rs429358	0.2461
4	APP	rs2154481	0.1917	NCK2	rs143080277	0.2120
5	INPP5D	rs35349669	0.1881	SUZ12P1	rs8093731	0.1904
6	SORT1	rs141749679	0.1851	MAF	rs450674	0.1811
7	APP	rs2154482	0.1729	ACE	rs4277405	0.1789
8	AGRN	rs113020870	0.1707	CD2AP	rs10948363	0.1677
9	DAB1	rs117567026	0.1707	CD2AP	rs7767350	0.1668
10	NGFR	rs9908234	0.1698	CD2AP	rs9296559	0.1651
11	SIGLEC11	rs9304690	0.1636	LILRB2	rs1761461	0.1641
12	APOE	rs769450	0.1611	CD2AP	rs9349407	0.1639
13	WNT3	rs199515	0.1570	CD2AP	rs9473117	0.1633
14	APOE	rs7412	0.1543	ACE	rs6504163	0.1630
15	C2orf80,IDH1	rs6722000	0.1526	CD2AP	rs9369716	0.1601
16	RBCK1	rs1358782	0.1511	ANO3	rs61877058	0.1593
17	INPP5D	rs10933431	0.1353	PTK2B	rs73223431	0.1590
18	BIN1	rs4663105	0.1322	INPP5D	rs10933431	0.1508
19	IGH	rs7157106	0.1230	ADAM10	rs442495	0.1464
20	HAVCR2	rs6891966	0.1220	USP6NL/ECHDC3	rs7912495	0.1457
21	BIVM-ERCC5	rs76029744	0.1218	PTK2B	rs28834970	0.1408
22	BIN1	rs6733839	0.1171	FAM155A	rs75174938	0.1388
23	SLC24A4	rs12881735	0.1120	FERMT2	rs17125944	0.1256

No.	Posterior			Lateral temporal		
	Gene	SNP	Z	Gene	SNP	Z
24	SLC24A4	rs7401792	0.1117	ZNRF2	rs11974360	0.1228
25	CLEC16A	rs8055533	0.1105	FOXF1	rs16941239	0.1188
26	MAGI2	rs3807779	0.1103	BIN1	rs4663105	0.1173
27	COX7C	rs62374257	0.1081	APOE	rs1081105	0.1168
28	TNIP1	rs871269	0.1076	CCDC134	rs7364180	0.1165
29	SLC24A4/BIN3	rs10498633	0.1020	KCNF1,FLJ33534	rs1809136	0.1150
30	MME	rs16824536	0.1009	CD33	rs1354106	0.1147
31	PRDM7	rs56407236	0.0951	CD2AP	rs9381563	0.1133
32	EPC2	rs2121433	0.0924	SCIMP/RABEP1	rs7209200	0.1127
33	KL	rs648202	0.0916	EPHA1	rs10808026	0.1122
34	KAT8	rs59735493	0.0894	HLA-DRB1	rs2516049	0.1117
35	PLCG2	rs72824905	0.0892	ADAM10	rs602602	0.1108
36	ABCA1	rs1800978	0.0881	IGH	rs10131280	0.1100
37	DOC2A	rs1140239	0.0860	INPP5D	rs7597763	0.1077
38	RHOH	rs2245466	0.0835	TSPAN14	rs6586028	0.1070
39	MS4A6A	rs610932	0.0808	PICALM	rs3844143	0.1016
40	GLIS3	rs59860681	0.0789	FERMT2	rs17125924	0.1006
41	ABI3	rs616338	0.0788	CASS4	rs6014724	0.1002
42	BCKDK	rs889555	0.0782	BIN1	rs6733839	0.1002
43	SHARPIN	rs61732533	0.0759	CTNNA3	rs10996833	0.0979
44	GRN	rs5848	0.0745	ELAVL2	rs7047280	0.0972
45	ADAMTS1	rs2830489	0.0744	CPXM2	rs72631124	0.0950
46	CALHM1	rs2986018	0.0716	CR1	rs679515	0.0942
47	MAPT	rs8070723	0.0700	IL34	rs4985556	0.0882
48	MAPT	rs242557	0.0664	NTN5	rs2452170	0.0880
49	TMEM106B	rs5011436	0.0618	COX7C	rs62374257	0.0877
50	MAGI1	rs58687721	0.0614	CD33	rs3865444	0.0870
51	TMEM106B	rs13237518	0.0604	DENND2B (ST5)	rs34033747	0.0844
52	SCIMP	rs9916042	0.0597	BIN1	rs744373	0.0806
53	FOXF1	rs16941239	0.0546	INPP5D	rs35349669	0.0799
54	KCNMA1	rs80058374	0.0535	CR1	rs3818361	0.0758
55	LILRB2	rs587709	0.0520	SORL1	rs74685827	0.0749
56	C16orf95	rs4843559	0.0427	SPPL2A	rs8025980	0.0724
57	TPCN1	rs6489896	0.0404	APP	rs2154481	0.0695
58	SCIMP/RABEP1	rs7209200	0.0381	IFNLR1	rs4649197	0.0682
59	BIN1	rs744373	0.0370	CASS4	rs6069737	0.0653
60	CR1	rs6656401	0.0367	CASS4	rs6024870	0.0650

Supplementary Table S4. Top 60 SNPs (average z-scored dosage) for Aβ—Cortex-priority and Subcortex-priority.

No.	Cortex-priority			Subcortex-priority		
	Gene	SNP	Z	Gene	SNP	Z
1	HSD17B6,SDR9C7	rs4526799	0.0757	MYO15A	rs2242595	0.1107
2	GRN	rs5848	0.0730	PLCG2	rs72824905	0.0885
3	SORL1	rs74685827	0.0658	GLIS3	rs59860681	0.0821
4	SIGLEC11	rs9304690	0.0644	ANK3	rs7068231	0.0801
5	GRN	rs708382	0.0641	CCDC6	rs7902657	0.0718
6	ZNRF2	rs11974360	0.0637	SEC61G	rs76928645	0.0712
7	LILRB2	rs1761461	0.0610	BIN1	rs6733839	0.0708
8	TSPAN14	rs6586028	0.0606	EPHA1	rs7810606	0.0689

No.	Cortex-priority			Subcortex-priority		
	Gene	SNP	Z	Gene	SNP	Z
9	CTNNA3	rs10996833	0.0606	RBCK1	rs1358782	0.0650
10	DOC2A	rs1140239	0.0602	PICALM	rs3844143	0.0636
11	INPP5D	rs10933431	0.0598	C21orf33,ICOSLG	rs55708341	0.0588
12	CD33	rs3865444	0.0586	MAPT	rs8070723	0.0566
13	HAVCR2	rs6891966	0.0578	USP6NL/ECHDC3	rs7912495	0.0564
14	ZFPM2	rs34823616	0.0513	WNT3	rs199515	0.0530
15	CYP4V2	rs7377304	0.0490	TPCN1	rs6489896	0.0522
16	ABI3	rs616338	0.0481	BIN1	rs4663105	0.0521
17	TREM2	rs187370608	0.0455	ECHDC3	rs11257238	0.0510
18	LILRB2	rs587709	0.0449	EZH2	rs10245290	0.0498
19	APOE	rs7412	0.0437	EPHA1	rs10808026	0.0498
20	TREM2	rs75932628	0.0429	ANKH	rs112403360	0.0495
21	CLU	rs11136000	0.0411	HLA-DRB1/HLA-DRB5	rs9271192	0.0490
22	CLU	rs1532278	0.0404	EPHA1-AS1	rs3935067	0.0489
23	CLU	rs11787077	0.0404	ABCA7	rs12151021	0.0488
24	AGRN	rs113020870	0.0391	PTK2B	rs12679874	0.0481
25	HS3ST5	rs785129	0.0372	NCK2	rs115186657	0.0477
26	CLU	rs9331896	0.0367	ICA1	rs10952097	0.0440
27	C2orf80,IDH1	rs6722000	0.0359	NCK2	rs143080277	0.0440
28	CD33	rs1354106	0.0320	ALPK2	rs76726049	0.0435
29	IFNLR1	rs4649197	0.0319	MEF2C	rs190982	0.0429
30	SORT1	rs141749679	0.0312	BIN1	rs744373	0.0403
31	CUL1	rs11974639	0.0306	APP	rs2154481	0.0389
32	KAT8	rs59735493	0.0293	KCNF1,FLJ33534	rs1809136	0.0385
33	EPC2	rs2121433	0.0290	ADAMTS4	rs4575098	0.0381
34	SPI1	rs10437655	0.0287	HTN1	rs200028958	0.0366
35	CELF1	rs10838725	0.0281	TMTC1	rs10843457	0.0348
36	RIN3	rs12590654	0.0277	PICALM	rs561655	0.0335
37	BLNK	rs6584063	0.0274	ADAM10	rs602602	0.0333
38	FERMT2	rs17125944	0.0253	MAPT	rs242557	0.0322
39	DAB1	rs117567026	0.0248	INPP5D	rs7597763	0.0314
40	PREP	rs72938040	0.0241	ABCA7	rs3764650	0.0312
41	IL2RA	rs7072793	0.0241	SLC24A4	rs7401792	0.0306
42	SORL1	rs11218343	0.0238	IGH	rs10131280	0.0303
43	CR1	rs3818361	0.0237	PTK2B	rs28834970	0.0301
44	BIVM-ERCC5	rs76029744	0.0236	DTHD1,MIR4801	rs66837203	0.0295
45	MAF	rs450674	0.0233	SCIMP	rs7225151	0.0289
46	SLC24A4	rs12881735	0.0225	SLC2A4RG	rs6742	0.0273
47	CR1	rs6656401	0.0221	PTK2B	rs73223431	0.0265
48	CALHM1	rs2986018	0.0218	PARK2	rs2187213	0.0261
49	SSBP4	rs7258465	0.0217	HLA-DRB1	rs2516049	0.0233
50	IPMK	rs12570088	0.0216	DCC	rs28592006	0.0227
51	ACE	rs4277405	0.0215	MS4A4A	rs1582763	0.0214
52	ADCY8,EFR3A	rs13260032	0.0214	COX7C	rs62374257	0.0211
53	KL	rs648202	0.0209	NCR2	rs6922617	0.0210
54	SCIMP	rs9916042	0.0205	ABI3	rs28394864	0.0207
55	PLEKHA1	rs7908662	0.0205	EPDR1	rs6966331	0.0206
56	SLC24A4/BIN3	rs10498633	0.0201	IGH	rs7157106	0.0179
57	CR1	rs679515	0.0197	SHARPIN	rs61732533	0.0176
58	QRFPR	rs6821123	0.0192	MME	rs61762319	0.0175
59	CSMD1	rs75778595	0.0186	GMNC	rs9877502	0.0172

No.	Cortex-priority			Subcortex-priority		
	Gene	SNP	Z	Gene	SNP	Z
60	CASS4	rs7274581	0.0181	ADAM10	rs442495	0.0171

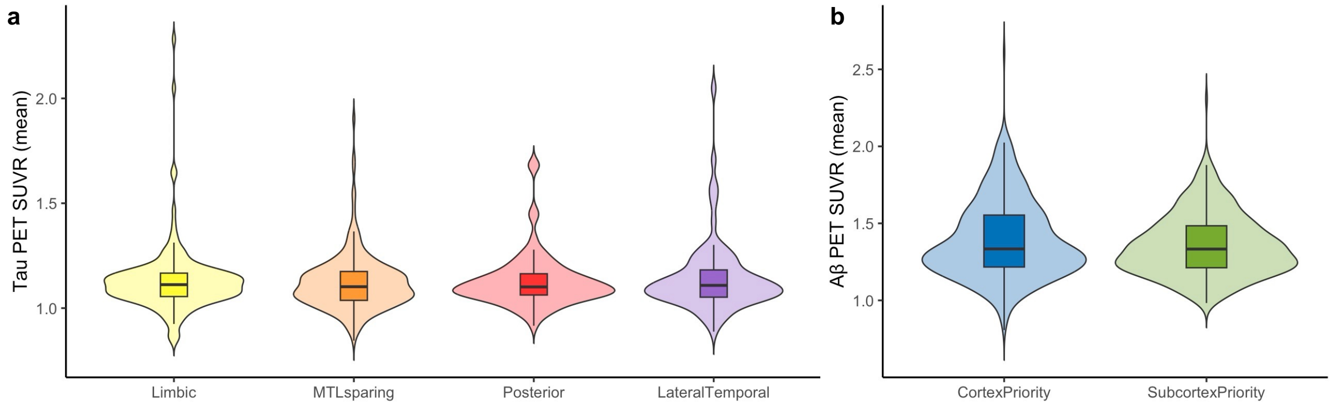

Supplementary Figure S1: (a) Violin plots showing the distribution of subject-level mean tau PET SUVR across four data-driven tau subtypes: Limbic, MTL-sparing, Posterior, and Lateral temporal. (b) Violin plots showing the distribution of subject-level mean Aβ PET SUVR across cortex-priority and subcortex-priority subtypes. Subtypes were identified using the SCCA-clustering approach applied to PET SUVR and risk allelic dosage from SNP genotypes. Violin shapes depict the kernel density of the distribution, and overlaid boxplots indicate the median (horizontal line) and interquartile range (box boundaries), with whiskers representing 1.5× the interquartile range.

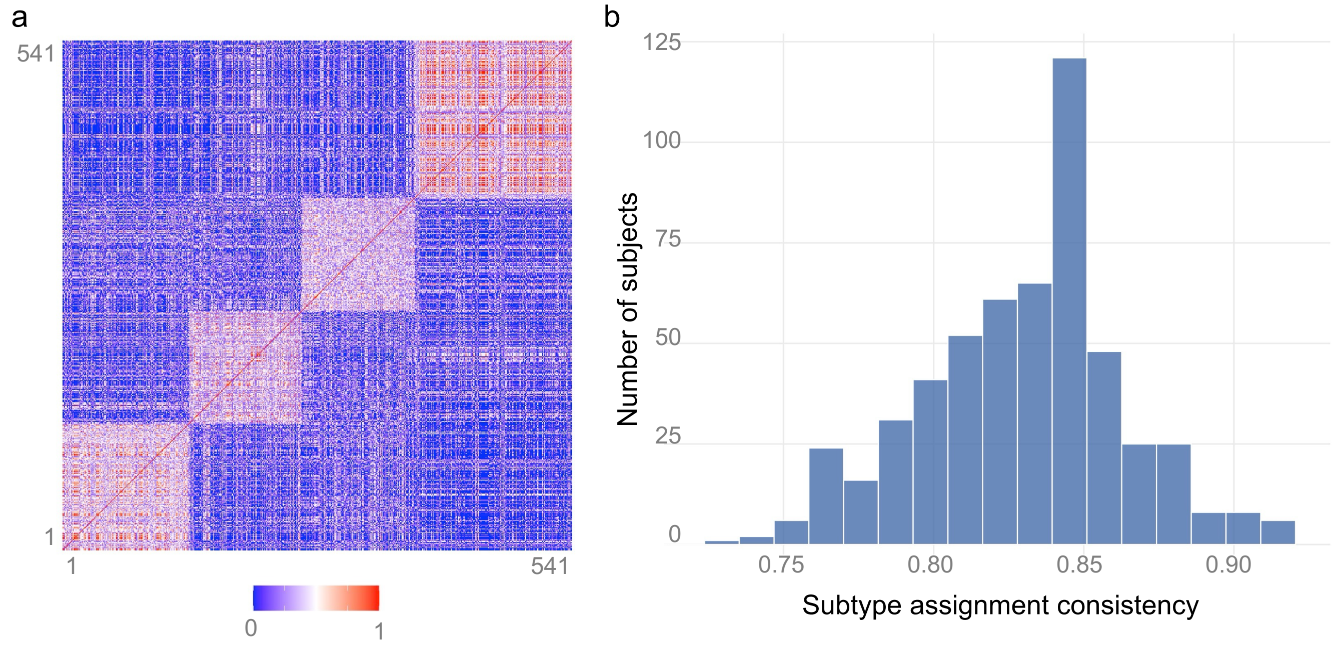

Supplementary Figure S2: (a) Consensus co-assignment matrix across 100 bootstrap repetitions, shown after sorting subjects by their baseline subtype. Brighter blocks along the diagonal indicate groups of individuals who consistently clustered together across resampled datasets, whereas lower co-assignment values near block boundaries reflect subjects whose tau patterns lie between subtype prototypes. (b) Distribution of per-subject subtype assignment consistency across the same 100 bootstrap repetitions. Most individuals were assigned to their predominant subtype in approximately 75%-90% of runs, indicating strong reproducibility of the tau subtype assignments under data resampling.

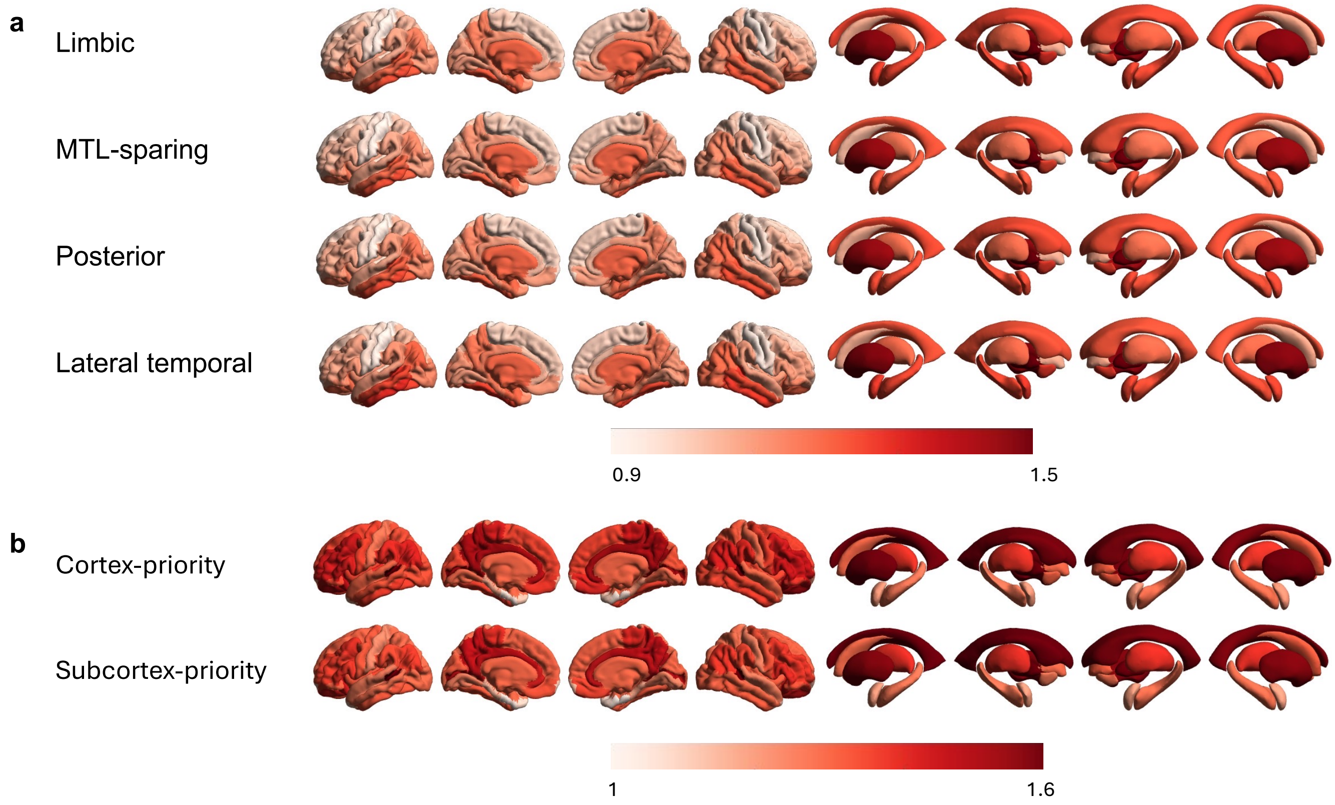

Supplementary Figure S3: (a) Tau PET SUVR maps for four data-driven tau subtypes (Limbic, MTL-sparing, Posterior, Lateral temporal) identified using the SCCA-clustering approach. (b) Aβ PET SUVR maps for two Aβ subtypes (cortex-priority, subcortex-priority) were identified using the same approach. For each participant, mean SUVR values were calculated within ROIs corresponding to each PET modality and averaged within each subtype. Color scales represent raw mean SUVR values to provide a direct visualization of absolute tracer uptake. Because these maps are based on unnormalized SUVRs, visual differences between subtypes may appear more subtle compared to the normalized z-score maps.

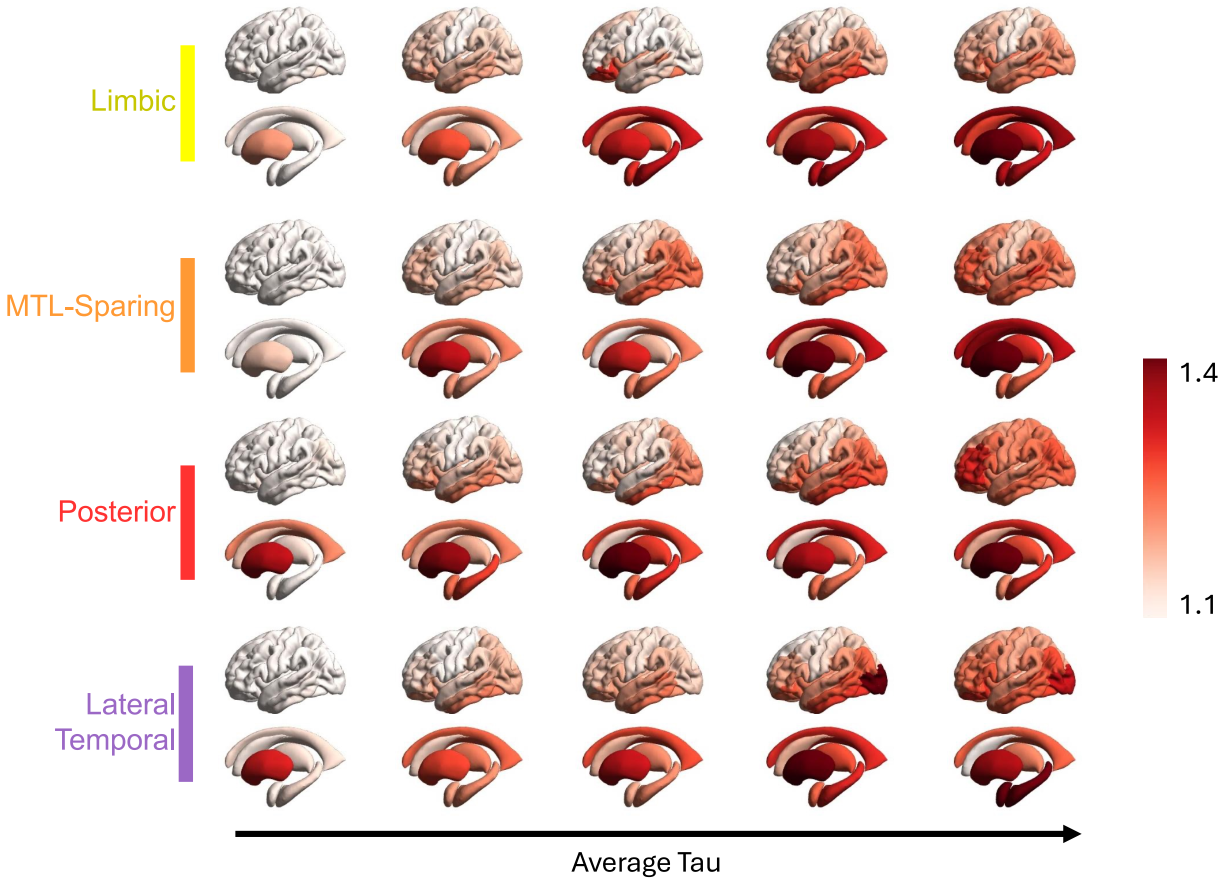

Supplementary Figure S4: Individual tau PET SUVR brain surface maps illustrating variation in each subtype. For each tau subtype (Limbic, MTL-sparing, Posterior, Lateral temporal), subjects were ranked by overall mean tau SUVR within the subtype; five were sampled at roughly equal intervals and are shown left-to-right in ascending average tau. For each subject, cortical surfaces (left) and subcortical renderings (right) display region-wise mean SUVR using a common color scale (e.g., 1.1-1.4 SUVR) to enable direct comparison. These examples are not intended as a formal stage sequence; instead, they illustrate that the four subtypes differ in spatial patterns rather than overall tau burden.

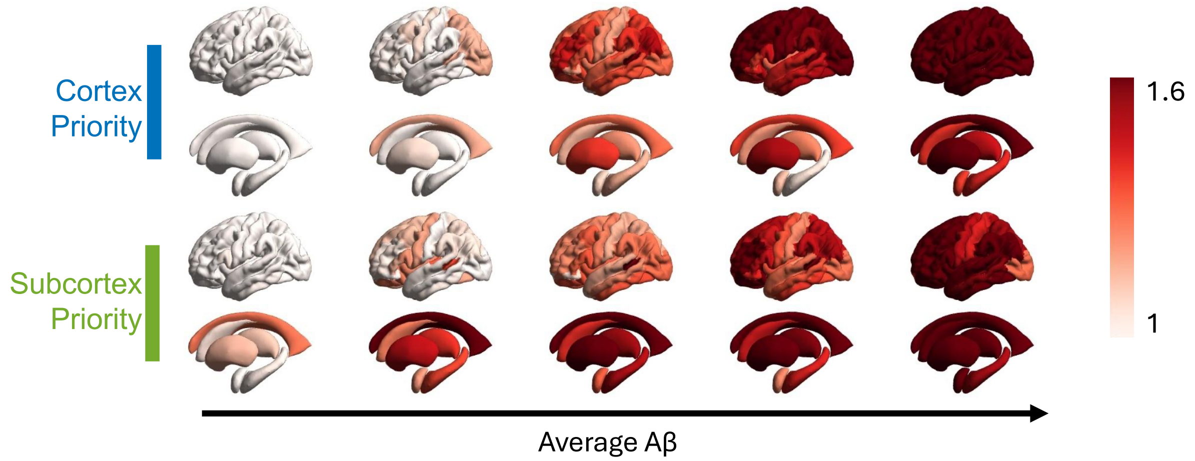

Supplementary Figure S5: Average Aβ PET SUVR brain surface maps for each subtype. Two Aβ subtypes (cortex-priority and subcortex-priority) were identified. For each subtype, cortical surfaces (left) and subcortical surfaces (right) display region-wise mean SUVR using a common color scale (1.0-1.4 SUVR) to enable direct comparison. These maps illustrate overall uptake distributions within each subtype, confirming that the subtypes are defined by their spatial characteristics rather than by differences in global Aβ burden.

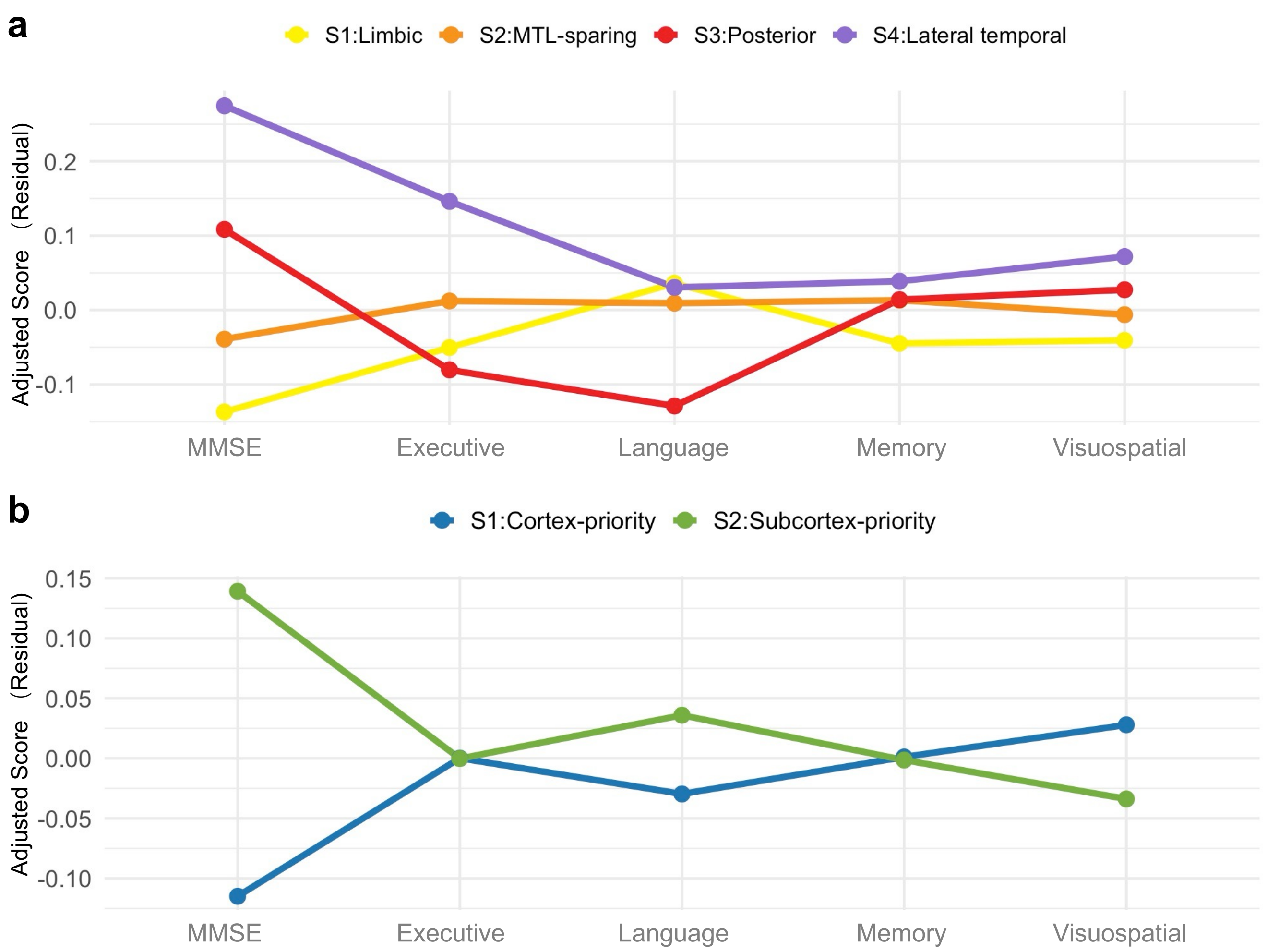

Supplementary Figure S6: Covariate-adjusted cognitive profiles for tau PET (top) and Aβ PET (bottom) subtypes. Plots show the mean residual cognitive scores across five domains (MMSE, executive, language, memory, and visuospatial) for each subtype. Residual scores were derived from linear regression models that adjusted each cognitive measure for diagnosis (CN/MCI/AD), age, and years of education. Thus, the plotted values represent subtype-related cognitive differences after removing variance attributable to clinical stage and demographic factors.

References

Allen, M., Kachadoorian, M., Quicksall, Z., Zou, F., Chai, H. S., Younkin, C., Crook, J. E., Pankratz, V. S., Carrasquillo, M. M., Krishnan, S., Nguyen, T., Ma, L., Malphrus, K., Lincoln, S., Bisceglio, G., Kolbert, C. P., Jen, J., Mukherjee, S., Kauwe, J. K., ... Ertekin-Taner, N. (2014). Association of MAPT haplotypes with Alzheimer's disease risk and MAPT brain gene expression levels. *Alzheimers Res Ther*, *6*(4), 39.

Alzgene website. (n.d.). Retrieved March 2, 2024, from http://www.alzgene.org/TopResults.asp

Bellenguez, C., kali, F., Jansen, I. E., Kleineidam, L., Moreno-Grau, S., Amin, N., Naj, A. C., Campos-Martin, R., Grenier-Boley, B., Andrade, V., Holmans, P. A., Boland, A., Damotte, V., van der Lee, S. J., Costa, M. R., Kuulasmaa, T., Yang, Q., de Rojas, I., Bis, J. C., ... Yang, Y. (2022). New insights into the genetic etiology of Alzheimer's disease and related dementias. *Nat Genet*, *54*(4), 412–436.

Chen, J., Yu, J. T., Wojta, K., Wang, H. F., Zetterberg, H., Blennow, K., Yokoyama, J. S., Weiner, M. W., Kramer, J. H., Rosen, H., Miller, B. L., Coppola, G., & Boxer, A. L. (2017). locus influencing human plasma tau levels. *Neurology*, *88*(7), 669–676.

Cruchaga, C., Kauwe, J. S., Harari, O., Jin, S. C., Cai, Y., Karch, C. M., Benitez, B. A., Jeng, A. T., Skorupa, T., Carrell, D., Bertelsen, S., Bailey, M., McKean, D., Shulman, J. M., De Jager, P. L., Chibnik, L., Bennett, D. A., Arnold, S. E., Harold, D., ... Drost, D. (2013). GWAS of cerebrospinal fluid tau levels identifies risk variants for Alzheimer's disease. *Neuron*, *78*(2), 256–268.

Du, L., Liu, K., Yao, X., Risacher, S. L., Han, J., Saykin, A. J., Guo, L., & Shen, L. (2020). Detecting genetic associations with brain imaging phenotypes in Alzheimer's disease via a novel structured SCCA approach. *Med Image Anal*, *61*, 101656.

Franzmeier, N., Rubinski, A., Neitzel, J., Ewers, M., Weiner, M. W., Aisen, P., Petersen, R., Jack, C. R., Jagust, W., Trojanowski, J. Q., Toga, A. W., Beckett, L., Green, R. C., Saykin, A. J., Morris, J., Shaw, L. M., Khachaturian, Z., Sorensen, G., Kuller, L., ... Furst, A. J. (2019). The BIN1 rs744373 SNP is associated with increased tau-PET levels and impaired memory. *Nat Commun*, *10*(1), 1766.

Genin, E., Hannequin, D., Wallon, D., Sleegers, K., Hiltunen, M., Combarros, O., Bullido, M. J., Engelborghs, S., De Deyn, P., Berr, C., Pasquier, F., Dubois, B., Tognoni, G., vet, N., Brouwers, N., Bettens, K., Arosio, B., Coto, E., Del Zompo, M., ... Campion, D. (2011). APOE and Alzheimer disease: a major gene with semi-dominant inheritance. *Mol Psychiatry*, *16*(9), 903–907.

Guerreiro, R., Wojtas, A., Bras, J., Carrasquillo, M., Rogaeva, E., Majounie, E., Cruchaga, C., Sassi, C., Kauwe, J. S., Younkin, S., Hazrati, L., Collinge, J., Pocock, J., Lashley, T., Williams, J.,

Lambert, J. C., Amouyel, P., Goate, A., Rademakers, R., ... Weale, M. (2013). TREM2 variants in Alzheimer's disease. *N Engl J Med*, *368*(2), 117–127.

Jansen, I. E., Savage, J. E., Watanabe, K., Bryois, J., Williams, D. M., Steinberg, S., Sealock, J., Karlsson, I. K., gg, S., Athanasiu, L., Voyle, N., Proitsi, P., Witoelar, A., Stringer, S., Aarsland, D., Almdahl, I. S., Andersen, F., Bergh, S., Bettella, F., ... Posthuma, D. (2020). Author Correction: Genome-wide meta-analysis identifies new loci and functional pathways influencing Alzheimer's disease risk. *Nat Genet*, *52*(3), 354.

Jansen, I. E., van der Lee, S. J., Gomez-Fonseca, D., de Rojas, I., Dalmasso, M. C., Grenier-Boley, B., Zettergren, A., Mishra, A., Ali, M., Andrade, V., Bellenguez, C., Kleineidam, L., kali, F., Sung, Y. J., í, N., Vromen, E. M., Wightman, D. P., Alcolea, D., Alegret, M., ... van der Flier, W. (2022). Genome-wide meta-analysis for Alzheimer's disease cerebrospinal fluid biomarkers. *Acta Neuropathol*, *144*(5), 821–842.

Kim, H. R., Lee, T., Choi, J. K., & Jeong, Y. (2021). on Neurodegeneration. *Alzheimer Dis Assoc Disord*, *35*(2), 114–120.

Kim, S., Swaminathan, S., Shen, L., Risacher, S. L., Nho, K., Foroud, T., Shaw, L. M., Trojanowski, J. Q., Potkin, S. G., Huentelman, M. J., Craig, D. W., DeChairo, B. M., Aisen, P. S., Petersen, R. C., Weiner, M. W., Saykin, A. J., Jack, C. R., Jagust, W., Toga, A. W., ... Mintzer, J. (2011). Genome-wide association study of CSF biomarkers Abeta1-42, t-tau, and p-tau181p in the ADNI cohort. *Neurology*, *76*(1), 69–79.

Kondo, T., Hara, N., Koyama, S., Yada, Y., Tsukita, K., Nagahashi, A., Ikeuchi, T., Ishii, K., Asada, T., Arai, T., Yamada, R., Inoue, H., Weiner, M. W., Aisen, P., Petersen, R., Jack, C. R., Jagust, W., Trojanowki, J. Q., Toga, A. W., ... Ishii, K. (2022). production using a large sample of individual iPSC lines derived from Alzheimer's disease patients. *Nat Aging*, *2*(2), 125–139.

Kunkle, B. W., Grenier-Boley, B., Sims, R., Bis, J. C., Damotte, V., Naj, A. C., Boland, A., Vronskaya, M., van der Lee, S. J., Amlie-Wolf, A., Bellenguez, C., Frizatti, A., Chouraki, V., Martin, E. R., Sleegers, K., Badarinarayan, N., Jakobsdottir, J., Hamilton-Nelson, K. L., Moreno-Grau, S., ... Pericak-Vance, M. A. (2019). , tau, immunity and lipid processing. *Nat Genet*, *51*(3), 414–430.

Magusali, N., Graham, A. C., Piers, T. M., Panichnantakul, P., Yaman, U., Shoai, M., Reynolds, R. H., Botia, J. A., Brookes, K. J., Guetta-Baranes, T., Bellou, E., Bayram, S., Sokolova, D., Ryten, M., Sala Frigerio, C., Escott-Price, V., Morgan, K., Pocock, J. M., Hardy, J., & Salih, D. A. (2021). A genetic link between risk for Alzheimer's disease and severe COVID-19 outcomes via the OAS1 gene. *Brain*, *144*(12), 3727–3741.

Naj, A. C., Jun, G., Beecham, G. W., Wang, L. S., Vardarajan, B. N., Buros, J., Gallins, P. J., Buxbaum, J. D., Jarvik, G. P., Crane, P. K., Larson, E. B., Bird, T. D., Boeve, B. F., Graff-Radford, N. R., De Jager, P. L., Evans, D., Schneider, J. A., Carrasquillo, M. M., Ertekin-Taner, N., ... Schellenberg,

G. D. (2011). Common variants at MS4A4/MS4A6E, CD2AP, CD33 and EPHA1 are associated with late-onset Alzheimer's disease. *Nat Genet*, *43*(5), 436–441.

Sims, R., van der Lee, S. J., Naj, A. C., Bellenguez, C., Badarinarayan, N., Jakobsdottir, J., Kunkle, B. W., Boland, A., Raybould, R., Bis, J. C., Martin, E. R., Grenier-Boley, B., Heilmann-Heimbach, S., Chouraki, V., Kuzma, A. B., Sleegers, K., Vronskaya, M., Ruiz, A., Graham, R. R., ... Schellenberg, G. D. (2017). Rare coding variants in PLCG2, ABI3, and TREM2 implicate microglial-mediated innate immunity in Alzheimer's disease. *Nat Genet*, *49*(9), 1373–1384.

Vacher, M., Porter, T., Villemagne, V. L., Milicic, L., Peretti, M., Fowler, C., Martins, R., Rainey-Smith, S., Ames, D., Masters, C. L., Rowe, C. C., Doecke, J. D., & Laws, S. M. (2019). Validation of a priori candidate Alzheimer's disease SNPs with brain amyloid-beta deposition. *Sci Rep*, *9*(1), 17069.

Van Cauwenberghe, C., Van Broeckhoven, C., & Sleegers, K. (2016). The genetic landscape of Alzheimer disease: clinical implications and perspectives. *Genet Med*, *18*(5), 421–430.

Wightman, D. P., Jansen, I. E., Savage, J. E., Shadrin, A. A., Bahrami, S., Holland, D., Rongve, A., rte, S., Winsvold, B. S., Drange, O. K., Martinsen, A. E., Skogholt, A. H., Willer, C., then, G., Bosnes, I., Nielsen, J. B., Fritsche, L. G., Thomas, L. F., Pedersen, L. M., ... Wilton, P. (2021). A genome-wide association study with 1,126,563 individuals identifies new risk loci for Alzheimer's disease. *Nat Genet*, *53*(9), 1276–1282.

Xue, Y. Y., Chen, Y. H., Lin, R. R., Huang, H. F., Wu, Z. Y., & Tao, Q. Q. (2022). Alzheimer's disease susceptibility locus in CD2AP is associated with increased cerebrospinal fluid tau levels in mild cognitive impairment. *Neurosci Lett*, *771*, 136419.

Yan, Q., Nho, K., Del-Aguila, J. L., Wang, X., Risacher, S. L., Fan, K. H., Snitz, B. E., Aizenstein, H. J., Mathis, C. A., Lopez, O. L., Demirci, F. Y., Feingold, E., Klunk, W. E., Saykin, A. J., Cruchaga, C., & Kamboh, M. I. (2021). Genome-wide association study of brain amyloid deposition as measured by Pittsburgh Compound-B (PiB)-PET imaging. *Mol Psychiatry*, *26*(1), 309–321.

Yokoyama, J. S., Wang, Y., Schork, A. J., Thompson, W. K., Karch, C. M., Cruchaga, C., McEvoy, L. K., Witoelar, A., Chen, C. H., Holland, D., Brewer, J. B., Franke, A., Dillon, W. P., Wilson, D. M., Mukherjee, P., Hess, C. P., Miller, Z., Bonham, L. W., Shen, J., ... Desikan, R. S. (2016). Association Between Genetic Traits for Immune-Mediated Diseases and Alzheimer Disease. *JAMA Neurol*, *73*(6), 691–697.
